# Associations between sex work laws and sex workers’ health: A systematic review and meta-analysis of quantitative and qualitative studies

**DOI:** 10.1371/journal.pmed.1002680

**Published:** 2018-12-11

**Authors:** Lucy Platt, Pippa Grenfell, Rebecca Meiksin, Jocelyn Elmes, Susan G. Sherman, Teela Sanders, Peninah Mwangi, Anna-Louise Crago

**Affiliations:** 1 Faculty of Public Health and Policy, London School of Hygiene & Tropical Medicine, London, United Kingdom; 2 Department of Epidemiology, Johns Hopkins Bloomberg School of Public Health, Baltimore, Maryland, United States of America; 3 Department of Criminology, University of Leicester, Leicester, United Kingdom; 4 Bar Hostess Empowerment and Support Programme, Nairobi, Kenya; 5 University of Toronto, Toronto, Ontario, Canada; Massachusetts General Hospital, UNITED STATES

## Abstract

**Background:**

Sex workers are at disproportionate risk of violence and sexual and emotional ill health, harms that have been linked to the criminalisation of sex work. We synthesised evidence on the extent to which sex work laws and policing practices affect sex workers’ safety, health, and access to services, and the pathways through which these effects occur.

**Methods and findings:**

We searched bibliographic databases between 1 January 1990 and 9 May 2018 for qualitative and quantitative research involving sex workers of all genders and terms relating to legislation, police, and health. We operationalised categories of lawful and unlawful police repression of sex workers or their clients, including criminal and administrative penalties. We included quantitative studies that measured associations between policing and outcomes of violence, health, and access to services, and qualitative studies that explored related pathways. We conducted a meta-analysis to estimate the average effect of experiencing sexual/physical violence, HIV or sexually transmitted infections (STIs), and condomless sex, among individuals exposed to repressive policing compared to those unexposed. Qualitative studies were synthesised iteratively, inductively, and thematically. We reviewed 40 quantitative and 94 qualitative studies. Repressive policing of sex workers was associated with increased risk of sexual/physical violence from clients or other parties (odds ratio [OR] 2.99, 95% CI 1.96–4.57), HIV/STI (OR 1.87, 95% CI 1.60–2.19), and condomless sex (OR 1.42, 95% CI 1.03–1.94). The qualitative synthesis identified diverse forms of police violence and abuses of power, including arbitrary arrest, bribery and extortion, physical and sexual violence, failure to provide access to justice, and forced HIV testing. It showed that in contexts of criminalisation, the threat and enactment of police harassment and arrest of sex workers or their clients displaced sex workers into isolated work locations, disrupting peer support networks and service access, and limiting risk reduction opportunities. It discouraged sex workers from carrying condoms and exacerbated existing inequalities experienced by transgender, migrant, and drug-using sex workers. Evidence from decriminalised settings suggests that sex workers in these settings have greater negotiating power with clients and better access to justice. Quantitative findings were limited by high heterogeneity in the meta-analysis for some outcomes and insufficient data to conduct meta-analyses for others, as well as variable sample size and study quality. Few studies reported whether arrest was related to sex work or another offence, limiting our ability to assess the associations between sex work criminalisation and outcomes relative to other penalties or abuses of police power, and all studies were observational, prohibiting any causal inference. Few studies included trans- and cisgender male sex workers, and little evidence related to emotional health and access to healthcare beyond HIV/STI testing.

**Conclusions:**

Together, the qualitative and quantitative evidence demonstrate the extensive harms associated with criminalisation of sex work, including laws and enforcement targeting the sale and purchase of sex, and activities relating to sex work organisation. There is an urgent need to reform sex-work-related laws and institutional practices so as to reduce harms and barriers to the realisation of health.

## Abstract 2

Lucy Platt and colleagues provide qualitative and quantitative evidence demonstrating the extensive harms associated with criminalisation of sex work.

## Author summary

**Why was this study done?:**

- To our knowledge there has been no evidence synthesis of qualitative and quantitative literature examining the impacts of criminalisation on sex workers’ safety and health, or the pathways that realise these effects.
- This evidence is critical to informing evidenced-based policy-making, and timely given the growing interest in models of decriminalisation of sex work or criminalising the purchase of sex (the latter recently introduced in Canada, France, Northern Ireland, Republic of Ireland, and Serbia).

**What did the researchers do and find?:**

- We undertook a mixed-methods review comprising meta-analyses and qualitative synthesis to measure the magnitude of associations, and related pathways, between criminalisation and sex workers’ experience of violence, sexual (including HIV and sexually transmitted infections [STIs]) and emotional health, and access to health and social care services.
- We searched bibliographic databases for qualitative and quantitative research, categorising lawful and unlawful police repression, including criminal and administrative penalties within different legislative models.
- Meta-analyses suggest that on average repressive policing practices of sex workers were associated with increased risk of sexual/physical violence from clients or other partners across 9 studies and 5,204 participants.
- Sex workers who had been exposed to repressive policing practices were on average at increased risk of infection with HIV/STI compared to those who had not, across 12,506 participants from 11 studies. Repressive policing of sex workers was associated with increased risk of condomless sex across 9,447 participants from 4 studies.
- The qualitative synthesis showed that in contexts of any criminalisation, repressive policing of sex workers, their clients, and/or sex work venues disrupted sex workers’ work environments, support networks, safety and risk reduction strategies, and access to health services and justice. It demonstrated how policing within all criminalisation and regulation frameworks exacerbated existing marginalisation, and how sex workers’ relationships with police, access to justice, and negotiating powers with clients have improved in decriminalised contexts.

**What do these findings mean?:**

- The quantitative evidence clearly shows the association between repressive policing within frameworks of full or partial sex work criminalisation—including the criminalisation of clients and the organisation of sex work—and adverse health outcomes.
- Qualitative evidence demonstrates how repressive policing of sex workers, their clients, and/or sex work venues deprioritises sex workers’ safety, health, and rights and hinders access to due process of law. The removal of criminal and administrative sanctions for sex work is needed to improve sex workers’ health and access to services and justice.
- More research is needed in order to document how criminalisation and decriminalisation interact with other structural factors, policies, and realities (e.g., poverty, housing, drugs, and immigration) in different contexts, to inform appropriate interventions and advocacy alongside legal reform.

## Introduction

Sex workers can face multiple interdependent health risks [1,2]. Between 32% and 55% of cisgender (cis) women working mostly in street-based sex work report experience of workplace violence in the past year [3]. Across diverse settings, both cis and transgender (trans) women and men in sex work are at increased risk of experiencing violence and homicide [4–6], HIV infection [7–9], chlamydia and gonorrhoea [10,11], and poorer mental health than their non-sex-working counterparts [12]. Yet there is considerable variation within sex-working populations [13,14]. The epidemiological context as well as social and structural factors and power relations reproduce inequalities within sex-working populations [2,3,8,9]. For example, cis women working in street-based sex work are more vulnerable to all these outcomes than those working in off-street settings [15,16]. Many vulnerabilities faced by sex workers are multiplicative, closely linked to poverty, substance use, disability, immigration, sexism, racism, transphobia, and homophobia [17].

Qualitative literature demonstrates how social policies and structural factors shape the health and welfare of sex workers. The ‘risk environment’ concept, developed to understand drug-related harms [18] and adapted to HIV and violence experienced by sex workers [19,20], examines different types (physical, social, economic, and political) and levels of environmental influence (micro and macro), in line with broader efforts to address structural determinants of health [21]. This concept has been used to demonstrate how policing, stigma, and inequalities interplay to shape sex workers’ vulnerability to HIV [22], violence [23], and lack of access to healthcare [24] and justice [25,26], and the potential for sex-worker-led interventions to challenge these harms [27]. Epidemiological evidence documents the associations between macro-structural factors (laws, housing and economic insecurity, migration, education, and stigma) and work environment and community factors (policing, work setting and conditions, autonomy, and access to health and peer-led services) and sex workers’ risk of violence and HIV transmission [2,3]. Criminalisation and repressive public health approaches to sex work (e.g., mandatory registration and HIV/sexually transmitted infection [STI] testing) have been shown to hinder the prevention of HIV, where the focus of interventions and research has been directed [28–30]. Conversely, mathematical modelling has estimated that decriminalisation of sex work could halve the incidence of HIV among sex workers and their clients over a 10-year period [2], and evidence from New Zealand indicates that sex workers in decriminalised settings report improved workplace safety, health and social care access, and emotional health [31,32].

Broadly, there are 5 legislative models used to manage, control, or regulate sex work (Table 1) [33]. Full criminalisation prohibits all organisational aspects of sex work and selling and buying sex. Partial criminalisation is where some aspects of sex work are penalised (e.g., soliciting sex in public for sex workers and/or clients, advertising services, collective working, or involvement of third parties). In 1999, Sweden criminalised the purchase, but not the sale, of sex, and various other countries have followed [34]. This ‘criminalisation of clients’ model typically retains laws against ‘brothel-keeping’, which may in practice also target sex workers working together. Regulatory models make the sale of sex legal in certain settings (e.g., in licensed brothels or managed zones) or under certain conditions (e.g., mandatory registration or HIV/STI testing) but illegal in other settings or for individuals who do not meet registration requirements or eligibility criteria (e.g., migrants, cis men and trans sex workers, or people living with HIV) [35]. Full decriminalisation, implemented in New Zealand in 2003, removes criminal penalties for adult sex work, emphasises enforcing criminal laws prohibiting violence and coercion, and regulates the sex industry through occupational health and safety standards [36]. All models criminalise coerced sex work and the involvement of minors, and almost all models—including decriminalisation in New Zealand—prohibit migrants without permanent residency from working legally or in a regulated environment. In practice the implementation of these models through bylaws and enforcement practices is complex, and varies between and within countries and even locally within cities [37,38].

**Table 1:** Sex work legislative models.

Legislative model	Broad definition	Countries operating these policies*
Full criminalisation	All aspects of selling and buying sex or organisation of sex work are prohibited.	South Africa, Sri Lanka, US^$^
Partial criminalisation	Organisation of sex work is prohibited, including working with others, running a brothel, involvement of a third party, or soliciting.	Canada (prior to 2014), India, UK (except Northern Ireland)
Criminalisation of purchase of sex	Often referred to as the sex-buyer model. Laws penalise sex workers working together (under third party laws), any aspect of participating in the sex trade as a third party, and buying sex.	Canada, France, Northern Ireland, Republic of Ireland, Norway, Serbia, Sweden
Regulatory models	Sale of sex is legal in licensed models and/or managed zones and is often accompanied by mandatory condom use, HIV/STI testing, or registration.	Australia (some states), Germany, Mexico, the Netherlands, Senegal
Full decriminalisation	All aspects of adult sex work are decriminalised, but condom use is legally required in some locations (i.e., New Zealand).	New Zealand

*This list summarises examples of countries where these models are implemented and represented in the review only, and is not exhaustive.

^$^There is some heterogeneity in the implementation of models within countries, including the US, where a legalised brothel system is in operation in Nevada.

The debate around sex work policy and legislation is highly polarised. Some argue that all sex work is itself gendered violence and should be repressed—a notion that underpins the criminalisation of sex workers’ clients [39,40]. Others argue that this fails to recognise the diversity of experience and identity in the sex industry and the possibility that financial reimbursement for sex between adults can be consensual [41]. At a time of increasing political interest in legislative reform [42–45], there is a critical need to bring together this evidence to inform policies that protect sex workers’ safety, health, well-being, and broader rights. We conducted a systematic review to synthesise evidence of the extent to which sex work laws and their enforcement affect sex workers’ safety, health and access to services, and the processes and pathways through which these effects occur, including in interaction with other macro-structural, community, and work environment factors.

## Methods

### Data extraction and quality assessment

Following a protocol with pre-specified search terms, we searched MEDLINE, CINAHL, PsychINFO, Web of Science, and Global Health for public health and social science literature on studies that combined 3 search domains: (1) sex work, AND (2) legislation OR policing, AND (3) health (physical or emotional, including violence/safety) OR access to services (including health, risk reduction, and social care/support). The complete search terms and review protocol are attached (S1 Text). Meta-analyses were not pre-specified, since they were subject to identifying sufficiently homogenous studies in relation to outcomes and definition of criminalisation.

Three authors screened the sources for inclusion, discussing any uncertainties within the team; a second person re-reviewed relevant sources when necessary. Quantitative data were extracted and analysed by LP and JE, and qualitative data synthesised by PG and RM. For qualitative and quantitative studies, we defined quality-related criteria adapted from the Critical Appraisal Skills Programme (CASP) [46] that papers had to fulfil in order to qualify for inclusion: methods and ethics processes described, appropriate study population clearly defined, and conclusions supported by study findings. Quantitative studies were further assessed according to appropriateness of study design, data collection methods, and analyses, using assessment approaches adapted from the Newcastle–Ottawa scale and CASP [46,47]. A full copy of the quality assessment process for the quantitative studies is available (S1 Table). For qualitative evidence, confidence in review findings was assessed according to CERQual guidance, taking account of methodological limitations, coherence, adequacy of data, and relevance of included studies (S2 Text) [48]. Methodological limitations were assessed using CASP guidelines for qualitative evidence.

### Definitions

We included studies with sex workers of all genders who currently or have ever exchanged sexual services for money, drugs, or other material goods. We included research on all models of sex work legislation and used the following definition of the criminalisation of sex work: ‘a model of intervention in which the criminal law is used to manage, control, repress, prohibit or otherwise influence the growth, instance or expression of prostitution’ [33]. We also included the use of non-criminal penalties to target sex workers, such as fines and displacement orders, including those that do not formally relate to sex work. Within the broad legislative models (Table 1), sex work legislation and policing was operationalised into 8 different categories of police exposure: (1) police repression on an environment in which sex work takes place (workplace raids, zoning restrictions, and displacement from usual working areas), (2) recent (within last year) arrest or prison, (3) past arrest or prison, (4) confiscation of condoms or needles or syringes, (5) extortion (giving police information, money, or goods to avoid arrest), (6) sexual or physical violence from police (negotiated or forced), (7) fear of police repression, and (8) registration as a sex worker at a municipal health authority. Where clear from included papers, we recorded data on gender using the terms ‘cis’ and ‘trans’ to refer to people who do and do not identify themselves with the gender they were assigned at birth, respectively. Conscious of cultural diversity in gender identities, we use the term ‘transfeminine’ to describe feminine-presenting trans populations that do not necessarily describe themselves as female/women [49]. We did not identify any papers that discussed the experiences of people who identify their gender as trans male/masculine or non-binary.

### Inclusion criteria

We included quantitative, qualitative, and mixed-methods studies published in English, Russian, or Spanish, and included data specific to the experiences of sex workers. We included papers that measured quantitative associations between criminalisation or decriminalisation of sex work, or repressive policing practices within these contexts, and the following outcomes: threatened or enacted violence, STIs, HIV, hepatitis B/C, overdose, stress, anxiety, depression, risk practices/management (e.g., working with others, reporting violence, condom use, sharing needles/syringes), and access to health/social care services (HIV/STI/hepatitis prevention, testing, and treatment; contraception; abortion; opioid substitution therapy and other drug/alcohol services; mental health and counselling; primary and secondary care; psychosocial support services; housing; and social security). We also included studies that reported qualitative data on the relationships between experiences of criminalisation or decriminalisation and policing and sex workers’ experiences of violence, safety, health, risk management, and/or accessing health or social care services, from the perspectives of sex workers themselves.

### Data synthesis

We synthesised estimates that adjusted for confounders to assess overall risk of experience of physical or sexual violence, HIV/STI, and condomless sex, stratified by the categories of repressive police activities described above. Where multiple policing practice exposures were presented in the same study, we selected independent estimates in an overall pooled estimate prioritising recent experience of arrest/prison and the most commonly occurring outcomes. Studies including sex workers of different genders were pooled together. We applied random effects models using the DerSimonian and Laird method for all analyses, allowing for heterogeneity between studies and converting all effect estimates into odds ratios (ORs) [50]. We examined heterogeneity with the *I*^2^ statistic. We conducted sub-group analyses to describe differences in experience of violence and condom use by partner type (client versus intimate partner/other) and by type of violence (physical versus sexual or sexual/physical combined). We conducted sensitivity analyses to look at overall associations between policing and our specified outcomes, excluding or pooling studies that did not adjust for confounders or reported only STI outcomes (self-reported and biological) or composite HIV/STI, and altering the priority choice of police exposure (from recent arrest/prison to other). We conducted a narrative synthesis of outcomes that were too heterogeneous to pool, including access to services (both mandatory and voluntary uptake of services), harms related to drug use, and emotional health. Studies that measured associations with registration at the municipal health department were also synthesised separately, since this policy was less comparable with all others that involved direct police action. All analyses were conducted using the metafor package in R version 3.4.1 and RStudio version 1.0.143 [51].

For qualitative studies, data were synthesised inductively, iteratively, and thematically. From the body of eligible papers we first focused on the ‘data-rich’ papers that contributed substantive or moderate data and analyses relevant to our research questions. Among the body of papers that had a limited focus on the topic, we then purposively sampled studies that reported on an under-represented population, setting, legislative model, or health issue of interest in this review [52] until no new themes emerged (thematic saturation). For the data-rich papers, we reviewed and wrote summaries of the results and discussion sections, inductively and iteratively drawing out author- and reviewer-identified themes and sub-themes. We then linked sub-themes and themes to 4 core categories, informed by concepts of structural, symbolic, and everyday violence that argue that mistreatment, stigma, exclusion, and ill health often result from intersecting inequalities that become institutionalised and normalised through policies, practices, and social norms [53]. We paid careful attention to the different levels and forms of environmental influence within risk environments [18]. Finally, we reviewed the less data-rich papers (relative to our research questions) against these emerging categories until they required no further refining. We summarise the core categories narratively with illustrative quotes (Box 1), drawing out findings that help to unpack the quantitative associations and their causal pathways. Within each category, we pay close attention to patterns by legislative model.

## Results

**Fig 1:**
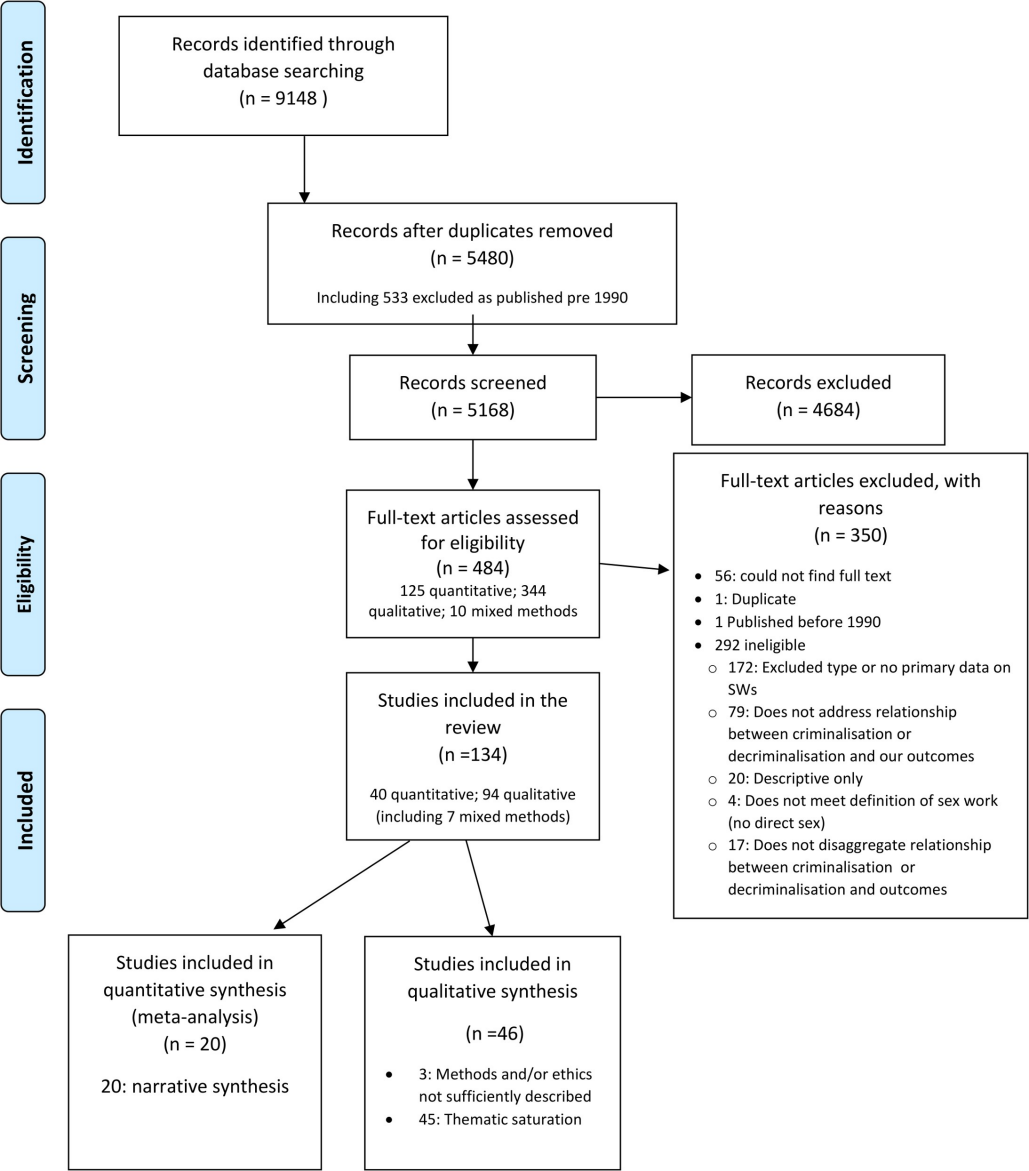
Flow chart of included qualitative and quantitative studies. SWs, sex workers.

From 9,148 papers identified, 134 studies met the inclusion criteria, resulting in 40 papers included in the quantitative synthesis, of which 20 were included in the meta-analysis and 20 in the narrative synthesis. A total of 94 met the inclusion criteria for the qualitative synthesis, of which 46 were included in the thematic analysis, 3 were excluded following quality assessment, and 45 were excluded when thematic saturation had been reached (Fig 1).

### Quantitative synthesis

#### Included quantitative studies

We identified 40 studies that measured the association between an aspect of police repression of sex workers or their clients and our outcomes of interest. The majority of the studies were cross-sectional (28) or serial cross-sectional (2); there were 9 prospective cohorts [27,54–61] and baseline data from 1 randomised control trial [62]. Studies were conducted in a variety of countries representing some but not all of the main sex work legislative models (Table 1). Partial criminalisation was represented in 10 studies in Canada, 6 studies in India, 3 studies in Russian Federation, 2 studies in Argentina, and 1 each in Côte D’Ivoire, Spain and UK. Full criminalisation was represented in 3 studies in Uganda, 2 studies in China, and 1 each in Iran, Rwanda, and South Korea. Regulation models were represented by 8 studies in Mexico. No quantitative studies examined the effects of the criminalisation of sex purchase in isolation, or the effects of decriminalisation. Outcomes reported included the following: sexual or physical violence (*n =* 10) [57–59,63–69], HIV and/or STI prevalence (*n =* 15) [54,60,63,67,70–78], condom use (*n =* 5) [71,74,78–82], access to services (*n =* 8) [56,61,63,71,80,83–85], aspects of drug use (*n =* 6) [27,46,62,63,66,86,87], and emotional ill health (*n =* 3) [55,60,88]. Two studies focused on the association between criminalisation and social and criminal justice factors including further extortion by the police or history of arrest [63], any contact with the criminal justice system, being a migrant, and unstable housing [60]. The majority of studies focused on cis women, with the exception of 6 that included trans women (*n =* 5) and cis men (*n =* 1) in Canada and Argentina [27,55,56,60,61,70]. Location of sex work was diverse across street and off-street settings. All studies reported an association between lawful or unlawful repressive police actions towards sex workers and outcomes, of which 21 adjusted for confounders. We synthesised 4 studies that reported an effect estimate associated with a mandatory registration separately [79,81,89,90] but considered lawful and unlawful repressive police activities within the regulatory system as part of the pooled analysis [63,72,91]. Three studies presented effect estimates associated with a policy change, STIs, and rushing negotiation with clients, and were also considered separately [57,77,92]. Twenty studies reported on outcomes relating to HIV/STI prevalence, violence, and condom use, on which our primary meta-analyses are based. Characteristics of all studies are summarised in Table 2.

**Table 2:** Summary of quantitative study characteristics and associations between lawful and unlawful police repression and sex workers’ experience of violence, condom use and HIV/STI outcomes, access to services, emotional health, and drug and alcohol use.

First author, year [reference] (quality appraisal)	Country	Study design and sample size	Population (setting)	Police exposure (time frame)	Percent	Outcome (time frame)	Unadjusted effect estimate	Adjusted effect estimate
**Partial criminalisation (organisation of sex work and soliciting)**								
Beattie, 2015 [71] (H)	India	Cross-sectional (serial), *n =* 5,792	Cis women (home, brothels)	Recent arrest (last year)	4.0	Chlamydia	2.4 (1.3–4.6)	1.8 (0.9–3.5)
						Gonorrhoea	4.5 (1.8–11.1)	2.7 (1.0–7.6)
						HIV	2.3 (1.5–3.5)	1.9 (1.2–3.1)
						Reactive syphilis	3.1 (1.9–5.1)	2.6 (1.5–4.1)
						No condom with last client for anal sex	0.5 (0.2–1.1)	0.8 (0.3–2.1)
						No condom with last regular partner	1.2 (0.8–1.7)	1.0 (0.6, 1.7)
						No condom with last sex client	0.7 (0.4–1.1)	0.6 (0.3–1.0)
						STI clinic in past 6 months	1.5 (0.9–2.5)	1.7 (1.0–3.0)
						Ever been to an non-governmental organisation meeting	0.9 (0.6–1.4)	1.2 (0.8–1.9)
						Member of a female sex worker collective	1.3 (0.9–2.0)	1.5 (0.9–2.2)
						Ever seen a peer educator	1.6 (0.6–4.4)	2.4 (0.8–7.1)
						Ever been to a drop-in centre	1.7 (1.1–2.7)	1.5 (0.9–2.4)
						Ever had an HIV test	0.9 (0.5–1.5)	1.2 (0.7–2.0)
Deering, 2013 [64] (H)	India	Cross sectional, *n =* 1,219	Cis women (street, home, brothels, dabhas [roadside cafes])	Recent arrest (last year)	5.7	Experienced physical or sexual violence by a client (1 year)		1.8 (1.0–3.3)
Erausquin, 2015 [74] (H)	India	Cross sectional (serial), *n =* 1,680	Cis women (home, highways, rural)	Confiscation of condoms (6 months)	7.6	STI symptoms*		2.4 (1.6–3.6)
					7.6	Money for sex without condom (6 months)		3.8 (2.6–5.6)
					7.6	Inconsistent condom use with clients (7 days)		1.7 (1.2–2.5)
				Extortion (gave gifts to police to avoid trouble in last 6 months)	14.8	STI symptoms*		2.4 (1.8–3.2)
					14.8	Money for sex without condom (6 months)		2.5 (1.8–3.5)
					14.8	Inconsistent condom use with clients (7 days)		1.6 (1.2–2.1)
				Police repression on sex work environment (raid in last 6 months)	36.1	STI symptoms*		2.2 (1.8–2.8)
					36.1	Money for sex without condom (6 months)		1.6 (1.2–2.1)
					36.1	Inconsistent condom use with clients (7 days)		1.1 (0.9–1.4)
				Recent arrest (6 months)	14.5	STI symptoms*		1.7 (1.3–2.3)
					14.5	Money for sex without condom (6 months)		1.5 (1.1–2.1)
				Recent arrest or prison	14.5	Inconsistent condom use with clients (7 days)		1.2 (0.9–1.6)
				Sexual or physical violence (had sex with police to avoid trouble)	11.1	STI symptoms*		2.2 (1.6–3.1)
						Money for sex without condom (6 months)		2.0 (1.4–2.9)
						Inconsistent condom use with clients (7 days)		1.2 (0.8–1.6)
Erausquin, 2011 [65] (H)	India	Cross-sectional, *n =* 835		Confiscation of condoms (6 months)	7.4	Sexual or physical violence from clients		5.6 (3.2–9.8)
				Extortion (gave gifts to police to avoid trouble in last 6 months)	12.0	Sexual or physical violence from clients		3.2 (2.0–5.0)
				Police repression on sex work environment (raid in last 6 months)	26.8	Sexual or physical violence from clients		4.6 (3.2–6.8)
				Recent arrest (6 months)	12.0	Sexual or physical violence from clients		7.1 (4.4–11.4)
				Sexual or physical violence (had sex with police to avoid trouble)	10.9	Sexual or physical violence from clients		3.1 (1.9–4.9)
Patel, 2015 [88] (H)	India	Cross sectional, *n =* 1,986	Cis women (street, home, brothel)	Ever experienced arrest/prison	N/A	Emotional ill health (depression defined through PHQ-2 scale)		1.6 (1.1–2.4)
Punyam, 2012 [84] (H)	India	Cross sectional, *n =* 1,986	Cis women (street, home)	Ever experienced arrest/prison	14.9	Emotional ill health (depression defined through PHQ-2 scale)		1.1 (0.8–1.4)
				Physical violence from police (police informed a friend/relative about sex work arrest)	44.6	Emotional ill health (depression defined through PHQ-2 scale)		1.8 (0.9–3.7)
Pando, 2013 [78] (H)	Argentina	Cross sectional, *n =* 1,255	Cis women (street, private off street)	Ever experienced arrest/prison because of sex work	45.4	HIV	4.4 (1.6–12.0)	1.8 (1.1–3.0)
						*Treponema pallidum*	2.1 (1.6–2.8)	1.5 (1.2–1.7)
						Irregular (not always) use of condoms with client	1.9 (1.3–2.7)	1.1 (0.9–1.4)
						Irregular (not always) use of condoms with partner	1.3 (0.9–2.0)	1.0 (0.8–1.3)
Avila, 2017 [70] (M)	Argentina	Cross-sectional, *n =* 273	Trans women	Ever experienced arrest	67.9	HIV	1.42 (0.82–2.47)	NS
						*Treponema pallidum*	2.4 (1.39–4.17)	NS
Platt, 2011 [67] (H)	UK	Cross sectional, *n =* 268	Cis women (massage saunas, flat, independent)	Ever experienced arrest/prison	20.2	STI/HIV^$^	1.3 (0.5–3.5)	2.0 (0.6–7.2)
						Physical violence^&^ from clients (12 months)	2.0 (1.1–3.9)	2.6 (1.1–5.7)
Estebanez, 1998 [75] (H)	Spain	Cross sectional, *n =* 2,914	Cis women (street, highway, bar, hotel/pension)	Ever experienced prison	15.9	HIV		1.1 (0.3–4.2)
		Cross-sectional, *n =* 261	Cis women who inject drugs	Ever experienced prison	8.4	HIV		1.7 (0.9–3.5)
Argento, 2015 [27] (H)	Canada	Prospective cohort, *n =* 692	Cis and trans women (street, bars, brothels)	Sexual or physical violence (harassment with and without arrest)	N/A	Use of non-prescription opioids (6 months)	2.4 (1.9–3.0)	1.8 (1.4–2.3)
Shannon, 2008 [85] (M)	Canada	Cross sectional, *n =* 198	Cis women (street)	Police repression on sex work environment (avoidance of healthcare access or harm reduction services due to violence [recent] and policing [presence and harassment])		Availability of health services and syringe availability		6.5 (4.0–10.6)
Shannon, 2009 [59] (H)	Canada	Prospective cohort, *n =* 205	Cis women	Police repression on sex work environment (moved working areas)	44.4	Being pressured by a client into unprotected vaginal or anal intercourse (6 month)	3.3 (1.4–7.6)	3.1 (1.4–7.4)
				Police repression on sex work environment (zoning restriction due to solicitation or drug charges)	8.8	Being pressured by a client into unprotected vaginal or anal intercourse (6 month)	3.4 (1.3–9.2)	3.4 (1.2–5.0)
Shannon, 2009 [58] (H)	Canada	Prospective cohort, *n =* 237	Cis women (street)	Confiscation of drug use paraphernalia (without arrest)		Clients perpetrated sexual or physical violence	1.3 (0.9–2.2)	N/A
						Forced to have sex (penetrative) against your will by someone** (6 month)	1.2 (0.3–2.0)	N/A
					N/A	Physically abused by someone** (6 month)	2.0 (1.2–3.1)	1.5 (1.0–2.4)
				Police repression on sex work environment (moved away from main streets)		Sexual or physical violence from client	2.2 (1.4–3.4)	2.1 (1.3–3.6)
						Forced to have sex (penetrative) against your will by someone** (6 month)	1.4 (0.9–2.3)	N/A
					N/A	Physically abused by someone** (6 month)	1.8 (0.9–3.0)	N/A
				Sexual or physical violence (assault)		Sexual or physical violence from client	4.2 (2.3–7.4)	3.4 (2.0–6.0)
						Forced to have sex (penetrative) against your will by someone** (6 months)	3.1 (1.6–6.0)	2.6 (1.3–5.2)
					N/A	Physically abused by someone** (6 months)	2.6 (0.9–3.8)	2.2 (0.8–3.6)
Socias, 2015 [60] (H)	Canada	Prospective cohort, *n =* 720	Cis and trans women (street, massage brothel)	Recent prison (6 months)¥	41.9	HCV infected	1.6 (1.1–2.2)	
					11.3	HIV infected	1.3 (0.8–2.0)	
						Injection drug use	2.1 (1.5–2.8)	
						Heavy drinking (≥ 4 drinks per day)	2.4 (1.5–3.8)	2.0 (1.2–3.0)
						Not born in Canada	11.1 (4.9–25.3)	3.3 (1.3–8.5)
						Unstable housing	5.6 (3.4–9.1)	4.3 (2.2–8.6)
Goldenberg, 2017 [56] (H)	Canada	Prospective cohort, *n =* 66	Cis and trans women	Density of displacement due to policing, within 250 m of residence		ART interruptions (≥2 consecutive days where no ART was dispensed at each semi-annual visit)	1.02 (1.01–1.04)	1.0 (1.0–1.0)
				Density of police harassment		ART interruptions (≥2 consecutive days where no ART was dispensed at each semi-annual visit)	1.01 (1.00–1.02)	N/A
				Density of ‘red zone’/legal restrictions on work areas (within a 250-m buffer of one’s residential location)		ART interruptions (≥2 consecutive days where no ART was dispensed at each semi-annual visit)	1.34 (1.02–1.75)	1.30 (0.97–1.76)
				Density of combined spatial criminalisation measures		ART interruptions (≥2 consecutive days where no ART was dispensed at each semi-annual visit)	1.0 (1.0–1.0)	1.0 (1.0–1.0)
Landsberg, 2017 [92] (M)	Canada	Prospective cohort (3 combined), *n =* 259	Cis women	Enforcement guideline that sought to prioritise the safety of and prevent violence towards sex workers, but continue to arrest clients and third parties		Rushed client negotiation due to police presence (last 6 months) measured after introduction of policy compared to before (after 2013 versus before)	1.71 (1.08–2.72)	1.73 (1.03–2.90)
		*n =* 100	Men				0.81 (0.27–2.43)	NS
Duff, 2017 [55] (H)	Canada	Prospective cohort, *n =* 545	Cis and trans women	Police presence reported to affect where sex workers worked	31.0	Work stress, including job control, psychological demands, work social support, physical demands	0.42 (0.30–0.53)	0.26 (0.14–0.38)
Sou, 2017 [61] (H)	Canada	Prospective cohort, *n =* 742	Cis and trans women (street, sauna, brothel)	Police harassment including arrest (6 months)	39.4	Unmet health need^≡^	1.48 (1.13–1.94)	1.57 (1.15–2.13)
Prangnell, 2018 [57] (M)	Canada	Prospective cohort (3 combined), *n =* 259	Cis women who inject drugs (street, sauna, brothel)	Enforcement guideline that sought to prioritise the safety of and prevent violence towards sex workers, but continue to arrest clients and third parties		Physical, sexual violence (6 months)	1.72 (0.78–3.80)	1.09 (0.59–2.04)
				Stopped, searched, or arrested (last 6 months)	24.3	Physical, sexual violence (6 months)	3.24 (1.78–5.88)	2.42 (1.33–4.40)
Wirtz, 2015 [87] (H)	Russia	Cross sectional, *n =* 754	Cis women (street, hotel, sauna, station)	Police extortion—money, sex, or information	28.4	Injecting drug use (in last 6 months)		3.0 (1.5–5.9)
				Police extortion—money	22.8	Injecting drug use (in last 6 months)		2.2 (1.1–4.7)
				Police extortion—sex	5.0	Injecting drug use (in last 6 months)		3.2 (1.2–8.7)
				Police extortion—information	3.5	Injecting drug use (in last 6 months)		3.0 (0.7–12.8)
Odinokova, 2014 [66] (M)	Russia	Cross sectional, *n =* 896	Cis women (street, hotel)	Sexual or physical violence (sexual coercion in context of police contact in the last 12 months)	38.2	Rape during sex work (ever)		2.1 (1.5–3.0)
Decker, 2012 [73] (M)	Russia	Cross sectional, *n =* 147	Cis women (street, hotel, saunas, agency, salons)	Sexual or physical violence—subotnik^#^ (3 months)	36.6	Any STI^/HIV	N/A	2.5 (1.2–5.4)
Lyons, 2017 [68] (M)	Côte D’Ivoire	Cross-sectional, *n =* 466	Cis women	Ever experienced arrest	26.4	Ever experienced physical violence^∩^	2.96 (1.89–4.63)	2.79 (1.77–4.41)
				Ever experienced arrest	3.0	Ever experienced physical violence^∩^	2.23 (0.69–7.21)	N/A
				Ever been harassed or irritated by police because of sex work	31.2	Ever experienced physical violence^∩^	3.17 (2.07–4.81)	2.86 (1.85–4.41)
				Ever felt like the police refused protection because of sex work	24.1	Ever experienced physical violence^∩^	3.03 (1.90–4.83)	2.75 (1.71–4.44)
				Ever experienced arrest	26.4	Ever experienced sexual violence^∩^	2.62 (1.72–4.01)	2.60 (1.65–4.90)
				Ever experienced arrest	3.0	Ever experienced sexual violence^∩^	3.44 (1.06–11.13)	4.51 (1.23–16.46)
				Ever been harassed or irritated by police because of sex work	31.2	Ever experienced sexual violence^∩^	1.80 (1.86–4.19)	2.53 (1.68–3.90)
				Ever felt like the police refused protection because of sex work	24.1	Ever experienced sexual violence^∩^	3.14 (2.01–4.89)	2.98 (1.86–4.80)
**Full criminalisation (selling and buying sex illegal)**								
Qiao, 2014 [80] (H)	China	Cross sectional, *n =* 794	Cis women (street, salon, hotels)	Ever experienced arrest/prison	5.7	Inconsistent condom use with clients (1 month)	0.8 (0.4–1.5)	N/A
				Fear of police repression	39.9	Inconsistent condom use with clients (1 month)	1.9 (1.4–2.6)	1.6 (1.0–2.4)
				Ever experienced arrest/prison	5.7	HIV testing (1 year)	3.7 (1.8–7.6)	2.7 (1.2–6.2)
				Fear of police repression	39.9	HIV testing (1 year)	0.8 (0.5–0.9)	0. 8 (0.5–1.1)
				Ever experienced arrest/prison	5.7	HIV prevention service^^	5.6 (1.7–18.4)	4.6 (0.9–23.3)
				Fear of police repression	39.9	HIV prevention service ^^	0.6 (0.4–0.8)	0.4 (0.2–0.7)
Zhang, 2013 [82] (H)	China	Cross sectional, *n =* 720	Cis women (street, brothels, massage parlours)	Ever experienced arrest		Unprotected sex in the last sex act	N/A	2.5 (1.4–4.6)
Jung, 2017 [77] (M)	South Korea	Cross-sectional (serial), *n =* 2,009	Women (brothels)	Sex Trafficking Act introduced in 2005 that criminalised buying and selling sex and closed down brothels		*Treponema pallidum* (comparing 2008 [before policy came into effect] with 2014)		0.29 (0.16–0.52)
						Gonorrhoea (comparing 2008 [before policy came into effect] with 2014)		0.22 (0.66–0.723)
Shokoohi, 2018 [86] (M)	Iran	Cross-sectional, *n =* 1,295	Cis women (street, home)	Recent experience of prison (12 months)	7.5	Use of crystal methamphetamine (1 month)	2.51 (1.44–4.37)	0.86 (0.47–1.58)
Braunstein, 2012 [54] (M)	Rwanda	Cross sectional, *n =* 192	Cis women	Ever experienced prison	47.0	HIV prevalence	N/A	1.8 (1.3–2.6)
	Rwanda	Prospective cohort, *n =* 397		Ever experienced prison	38.0	HIV seroconversion	N/A	1.4 (0.5–3.8)
Erickson, 2015 [83] (H)	Uganda	Cross sectional, *n =* 400	Cis women	Fear of police exposure leading to rushed negotiations with clients	37.3	Dual contraceptive use	0.6 (0.4–0.9)	0.6 (0.4–1.0)
Goldenberg, 2016 [76] (H)	Uganda	Cross-sectional, *n =* 400	Cis women (bars, clubs, public places, highway)	Ever experienced prison	26.5	HIV	1.67 (1.06–2.64)	1.93 (1.17–3.20)
				Rushed client negotiation because of police presence (6 months)	37.3	HIV	0.99 (0.64–1.52)	N/A
Muldoon, 2017 [69] (H)	Uganda	Cross-sectional, *n =* 400	Cis women (bars, clubs, public places, highway)	Rushed client negotiation because of police presence (6 months)	37.3	Sexual or physical violence from clients (last 6 months)	2.28 (1.51–3.46)	1.61 (1.03–2.52)
**Regulation through registration in certain zones but public soliciting illegal**								
Pitpitan, 2016 [62] (H)	Mexico	RCT, *n =* 300	Cis women who inject drugs (street, bar)	Confiscation of needle/syringe	30	Injected with used needle/syringe	−0.51 (SE 0.25)	
Strathdee, 2011 [91] (H)	Mexico	Cross-sectional within RCT, *n =* 620	Cis women who inject drugs (street, bars, massage parlour)	Confiscation of syringes instead of arrest	29.0	HIV infection	2.4 (1.2–4.8)	2.4 (1.2–6.5)
				Extortion (bribes instead of arrest)	63.0	HIV infection	1.6 (0.7–3.5)	
Beletsky, 2013 [63] (H)	Mexico	Cross sectional, *n =* 624	Cis women who inject drugs (street)	Confiscation of syringes in last 6 months	48.0	Any STI (gonorrhoea, chlamydia	1.4 (1.0–1.9)	
						HIV infection	2.4 (1.1–5.1)	2.5 (1.1–5.8)
						Syphilis (based on titre ≥ 1:8)	1.5 (1.1–2.2)	
						Police requested sexual favours (6 months)	5.9 (4.0–8.6)	
						Sexually abused by police (6 months)	11.7 (6.3–22.0)	12.8 (6.6–24.2)
						Ever had an HIV test	1.5 (1.1–2.1)	
						Normally injected in public places	1.7 (1.3–2.4)	1.6 (1.1–2.4)
						Often/always injected with a client around in the last 6 months	0.7 (0.5–1.0)	0.6 (0.4–0.9)
						Groin injecting	1.9 (1.3–3.0)	1.8 (1.1–2.9)
						Police officer requested money (6 months)	18.6 (11.8–29.3)	
						Police officer forcibly took money (6 months)	11.8 (8.1–17.3)	
						Emotional ill health^+^	1.6 (1.1–2.1)	
				Extortion (bribes instead of arrest)	63.0	HIV prevalence	1.6 (0.7–3.5)	
Chen, 2012 [72] (H)	Mexico	Cross sectional, *n =* 200	Cis women (street, bar venues, truck routes)	Ever experienced arrest	28.6	STI symptoms	2.5 (1.1–5.3)	2.3 (1.0–5.0)
				Recent arrest (last year)	16.5	STI symptoms	2.2 (0.9–5.4)	
Gaines, 2013 [79] (H)	Mexico	Cross sectional, *n =* 181	Cis women (bar)	Registration at the Municipal Health Department	52.0	Free condoms available at venue	2.3 (0.8–6.5)	2.4 (0.9–6.1)
						In a bad financial situation	0.6 (0.3–1.1)	0.7 (0.3–1.6)
						Non-injection use of methamphetamines in the past month	0.2 (0.1–0.5)	0.3 (0.1–0.6)
						Ever tested for HIV	6.1 (2.6–14.2)	5.4 (2.3–12.5)
						Injected cocaine in the past month	0.1 (0.01–1.2)	0.1 (0.01–0.9)
Rusch, 2010 [89] (H)	Mexico	Cross-sectional, *n =* 331	Cis women (bar)	Registration at the Municipal Health Department	44.7	Working in a venue with high HIV/STI (syphilis) prevalence	0.4 (0.2–0.8)	0.5 (0.2–1.0)
Sirotin, 2010 [81] (M)	Mexico	Cross sectional, *n =* 187	Cis women (street, bar)	Registration at the Municipal Health Department	44.7	Any STI (syphilis, gonorrhoea, chlamydia, HIV)	0.4 (0.3–0.6)	NS
						Gonorrhoea	0.3 (0.1–0.7)	NS
						Chlamydia	0.8 (0.5–1.3)	NS
						Any positive syphilis titre > 1:1	0.3 (0.2–0.5)	NS
						HIV positive	0.4 (0.2–1.0)	NS
						Unprotected vaginal sex with clients in the past month (median percentage)	0.6 (0.3–1.1)	NS
						Ever been tested for HIV/AIDS	4.8 (2.9–7.8)	4.2 (2.3–7.5)
						Has clients who have ever injected drugs	0.5 (0.4–0.8)	NS
						Ever injecting drugs	0.2 (0.1–0.3)	NS
						Injected cocaine in the past month	0.1 (0.01–0.5)	0.1 (0.01–0.6)
Sirotin, 2010 [90] (M)	Mexico	Cross-sectional, *n =* 474	Cis women (street, bar)	Lack of registration at the Municipal Health Department	43.3	Unprotected sex	1.55 (0.94–2.57)	2.06 (1.21–3.50)
						Ever injected drugs	1.43 (1.05–1.93)	N/A

Quality appraisal definitions: H = high, M = moderate, L = low.

*STI symptoms in [74] defined as abdominal pain not relating to diarrhoea or menses, foul smelling vaginal discharge, pain while urinating, genital ulcers/sores, swelling in groin area, or itching in last 6 months. STI symptoms in [72] defined as having genital/anal warts, genital ulcers or sores, genital itching, or abnormal vaginal discharge in the past 6 months.

^$^STI/HIV defined as past infection with HIV or *Treponema pallidum* or acute infection with chlamydia or gonorrhoea [67].

^&^Physical violence defined as reporting 1 or more of the following: robbed, hit, beaten, threatened, attacked with a weapon, or kidnapped [67]

**Perpetrator of violence includes partner, pimp, dealer, police, security guard, stranger, or other but excludes clients.

¥Socias et al 2015: Recent prison is presented as the outcome in the original analysis but as temporal associations were not measured the outcomes and exposure variables have been inverted for the review in order to facilitate comparison.

^≡^Unmet health need defined as sometimes, occasionally, or never getting healthcare services when you need them versus always or usually getting them [61].

^#^Subotnik is defined as sex demanded by police in exchange for leniency towards pimps and female sex workers in past 3 months [73].

^Includes gonorrhoea, syphilis, and chlamydia [73].

^∩^Physical violence defined as ever having been violently pushed, shoved, slapped, hit, kicked, choked, or otherwise physically hurt. Sexual violence defined as ever having experienced forced sex through physical force, coercion, or penetration with an object against one’s will [68].

^^HIV prevention package included condom distribution, community-based methadone maintenance treatment and/or needle and syringe programme, and peer HIV/AIDS education [80].

^+^Emotional ill health defined as reported diagnosis of depression, post-traumatic stress disorder, anxiety, schizophrenia, borderline personality, attention deficit, or bipolar disorder within last 6 months [63].

HCV, hepatitis C virus; N/A, not available; NS, not significant; PHQ-2, Patient Health Questionnaire–2; RCT, randomised control trial; STI, sexually transmitted infection.

#### HIV and STI outcomes

**Fig 2:**
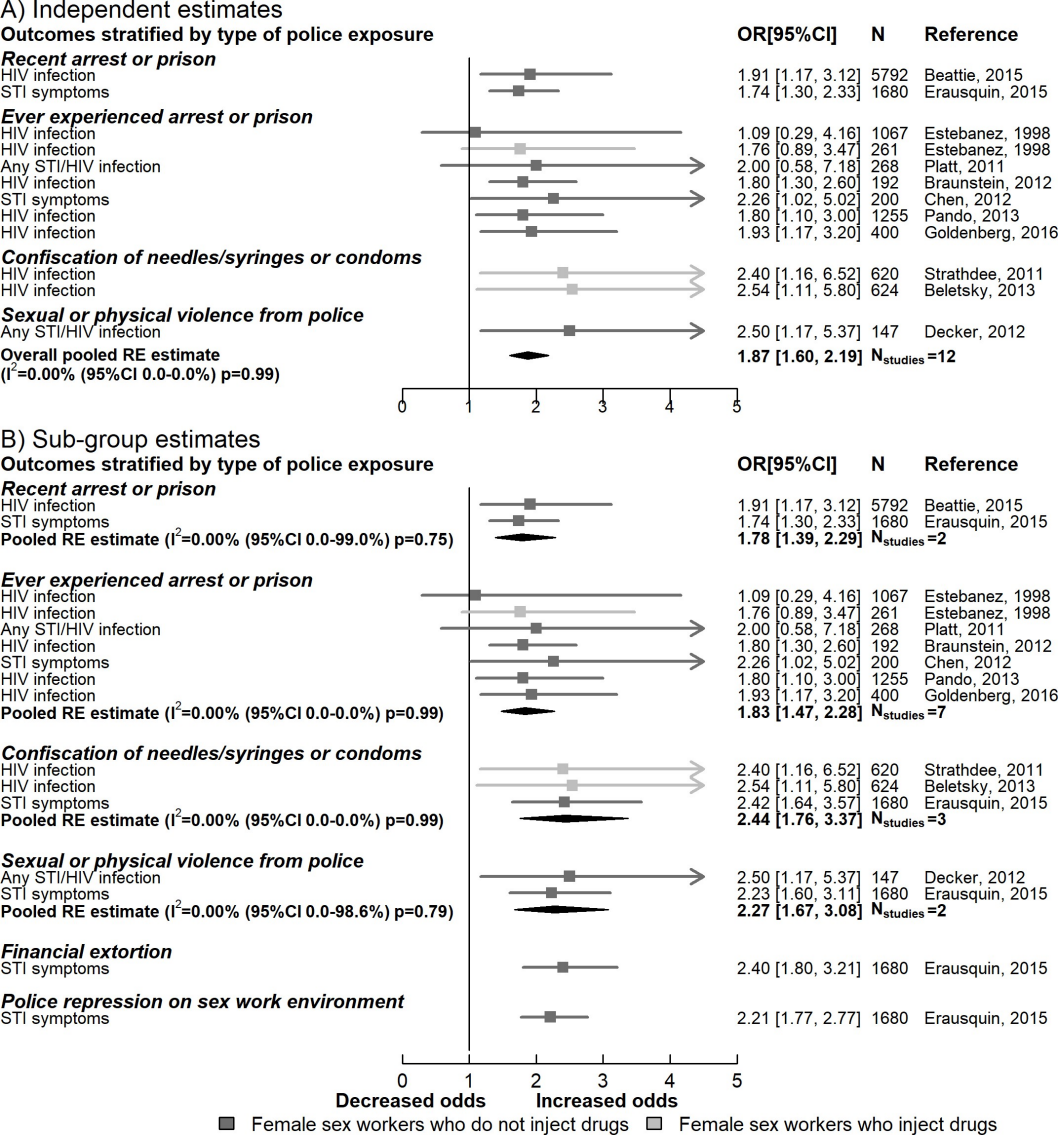
Meta-analyses summarising associations between repressive policing actions on HIV and sexually transmitted infections. RE, random effects; STI, sexually transmitted infection.

Meta-analysis of 12 independent multivariable estimates showed that any type of repressive police practice was associated with twice the odds of HIV/STI (12,506 participants, OR 1.87, 95% CI 1.60–2.19), with little heterogeneity between studies (*I*^2^ = 0.0%, 95% CI 0.0%–0.0%, *p =* 0.99). Sub-group analysis suggested that people who had their needles/syringes or condoms confiscated had higher odds of HIV/STIs than those who did not (2,924 participants, OR 2.44, 95% CI 1.76–3.37, *I*^2^ = 0.0%, 95% CI 0.0%–0.0%, *p =* 0.99). Sex workers who had experienced sexual or physical violence from police had higher odds of HIV/STI compared to those who had not (1,827 participants, OR 2.27 95% CI 1.67–3.08, *I*^2^ = 0.0%, 95% CI 0.0%–98.6%, *p =* 0.79) (Fig 2).

The overall effect estimate of repressive policing actions on HIV/STI outcomes was maintained across sensitivity analyses including those focusing on unadjusted estimates (OR 1.85, 95% CI 1.49–2.30, *I*^2^ = 14.0%, 95% CI 0.0%–81.1%, *p =* 0.32) (S1 Fig), those focusing on HIV outcomes only (OR 1.88, 95% CI 1.54–2.28, *I*^2^ = 0.0%, 95% CI 0.0%–0.0%, *p =* 0.98), and those excluding self-reported STI symptoms (OR 1.91, 95% CI 1.58–2.31, *I*^2^ = 0.0%, 95% CI 0.0%–0.0%, *p =* 0.99) (S4 Fig).

#### Violence

**Fig 3:**
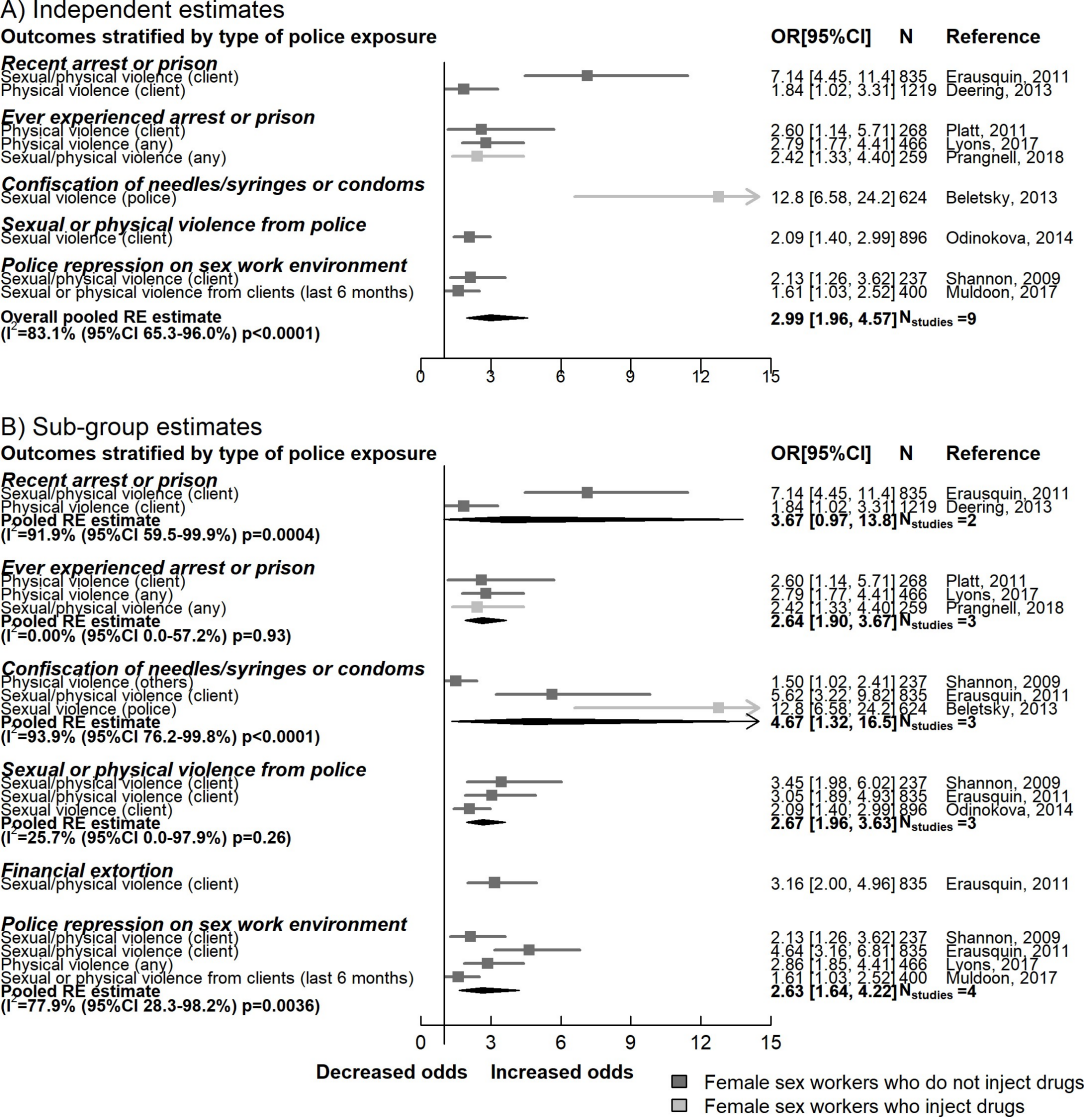
Meta-analyses summarising the association between repressive policing actions and sexual/physical violence from clients, intimate partners, and others. Shannon, 2009 refers to [58]. RE, random effects.

We pooled data from 9 studies that measured the association between repressive policing activities and experience of physical or sexual violence against sex workers by a range of perpetrators, including clients, intimate (sex) partners, and police. Random effects meta-analysis of 9 independent multivariable estimates showed that, overall, repressive policing was associated with substantially higher odds of any kind of violence (5,204 participants, OR 2.99, 95% CI 1.96–4.57), but with high heterogeneity (*I*^2^ = 83.1%, 95% CI 65.3%–96.0%, *p <* 0.001). Sub-group analysis suggested that those who had their needles/syringes or condoms confiscated had higher odds of violence than those who did not (1,696 participants, OR 4.67, 95% CI 1.32–16.54, *I*^2^ = 93.9%, 95% CI 76.2%–99.8%, *p <* 0.01) (Fig 3).

This overall association between police repression and violence increased slightly, but was still associated with substantially higher odds of violence, when all unadjusted estimates were pooled from 6 studies (OR 3.15, 95% CI 1.99–4.99, *I*^2^ = 78.7%, 95% CI 52.5%–97.4%, *p <* 0.001) (S2 Fig). Odds of experiencing physical or sexual violence by other people (defined as anyone other than paying clients, including the police) was higher for those who had experienced any type of repressive police activity compared to those who had not (OR 3.72, 95% CI 1.74–7.95, *I*^2^ = 84.1%, 95% CI 53.5%–99.0%, *p <* 0.001). Similarly, physical or sexual violence from clients was higher among those who had been exposed to repressive police activity compared to those who had not (OR 2.71, 95% CI 1.69–4.36, *I*^2^ = 80.4%, 95% CI 45.5%–96.3%, *p <* 0.001) (S4 Fig).

#### Condom use

**Fig 4:**
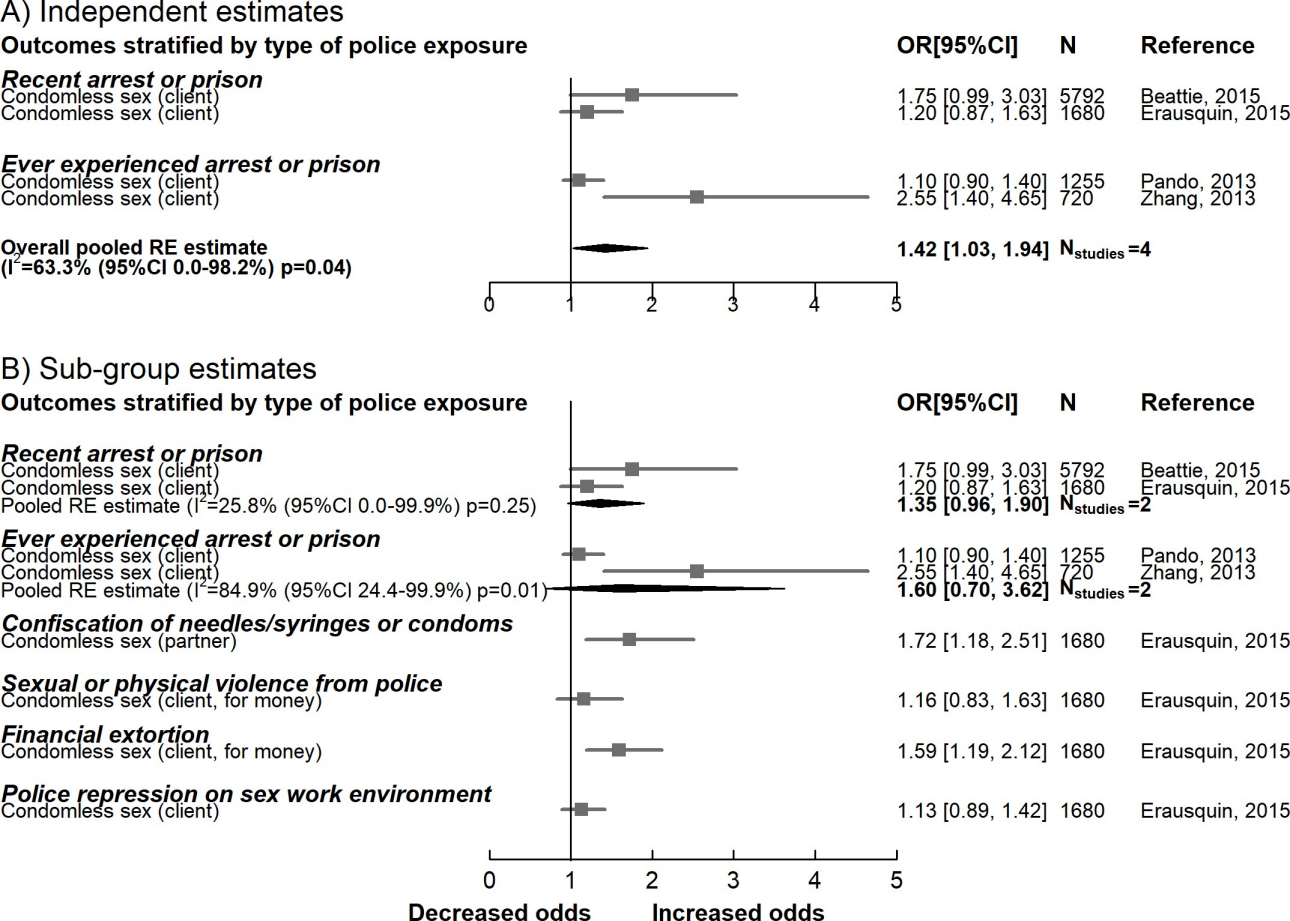
Meta-analyses summarising the association between repressive policing actions and condomless sex with clients and intimate partners. RE, random effects.

Five studies measured the association between repressive policing activities and condom use with both paying and non-paying partners. Meta-analysis of 4 independent multivariable estimates (9,447 participants) suggested that on average these practices were associated with increased odds of not using a condom (OR 1.42, 95% CI 1.03–1.94), with moderate heterogeneity across the studies (*I*^2^ = 63.34%, 95% CI 0.0%–98.2%, *p =* 0.04) (Fig 4).

The overall association between repressive policing activities and condom use increased when pooling unadjusted estimates from 2 studies (OR 1.76, 95% CI 1.30–2.38, *I*^2^ = 0.0%, 95% CI 0.0%–0.98%, *p =* 0.46) (S3 Fig). Sub-group analysis suggested that the odds of condomless sex with clients was higher following policing exposure (OR 1.42, 95% CI 1.03–1.94, *I*^2^ = 63.3%, 95% CI 0.0%–98.2%, *p =* 0.04) or when additional money was offered (OR 1.54, 95% CI 1.10–2.15, *I*^2^ = 66.7%, 0.0%–97.8%, *p =* 0.03). There was no difference in the odds of condomless sex with non-paying partners after police exposure (OR 1.0, 95% CI 0.80–1.24, *I*^2^ = 0.0%, 95% CI 0.0%–17.7, *p =* 0.97) (S4 Fig).

#### Access to services and mandatory testing

Five studies looked at the association between repressive policing activities and access to health and social care services. One study in India found that arrest in the last year was associated with increased odds of attendance at an STI clinic (OR 1.74, 95% CI 1.02–2.98, *p =* 0.04) [71]. Confiscation of needles/syringes in Mexico by the police was associated with increased odds of having an HIV test among sex workers who inject drugs (OR 1.49, 95% CI 1.09–2.05, *p-*value not reported) [63]. In Canada, fear of police and police harassment, including arrests, was associated with avoiding healthcare services among street-based cis women [85] and cis and trans women [61]. Geospatial analyses among the same population showed that a higher density of police enforcement practices (including displacement, legal restrictions of sex work areas, and police harassment) was associated with disrupted HIV treatment [56]. In Uganda, rushed negotiations with clients due to police presence was associated with less frequent dual contraceptive use (OR 0.65, 95% CI 0.42–1.00, *p =* 0.05) [83]. In a study in China, where HIV testing is mandatory following detention, history of arrest was associated with increased odds of having an HIV test or taking up HIV prevention interventions, but fear of arrest was associated with decreased odds of both HIV testing (OR 0.78, 95% CI 0.55–1.12, *p =* 0.18) and accessing prevention interventions (OR 0.39, 95% CI 0.22–0.68, *p <* 0.001) [80].

#### Emotional ill health

Three studies looked at indicators of emotional ill health. In India, cis female sex workers mostly working on the street who had been arrested had increased odds of major depression (defined through Patient Health Questionnaire–2) (OR 1.6, 95% CI 1.1–2.3, *p =* 0.05) compared to those who had not been arrested [88]. In Canada, recent incarceration was associated with poor emotional health outcomes among both cis and trans female sex workers in a univariable analysis (OR 1.55, 95% CI 1.12–2.14, *p <* 0.10) [60]. Among the same population, individuals who reported that the police had affected where they worked had increased work stress compared to those who did not report this [55].

#### Drug and alcohol use

Five studies examined the association between repressive policing practices and drug use including injecting drug use [60,66,86,87], the use of non-prescription opioids [27], and excessive alcohol drinking [60,66]. All of these studies showed a positive association between exposure to repressive policing practices and drug/alcohol use. One study among cis female sex workers in Mexico who inject drugs found a positive association between police confiscation of needles/syringes and injecting in public places (linked to increased risk of skin and soft tissue injuries but reduced risk of overdose) (OR 1.6, 95% CI 1.1–2.4, *p-*value not reported), as well as injecting in the groin area (linked to increased risk of overdose) (OR 1.9, 95% CI 1.2–2.9, *p-*value not reported), but reduced odds of injecting with clients (potentially linked to sharing needles/syringes but reduced risk of overdose) (OR 0.64, 95% CI 0.44–0.94, *p-*value not reported) [63]. Another study with the same population found that confiscation of needles/syringes was associated with lower safe injection self-efficacy at 8 months (−0.51, SE 0.25, *p =* 0.04) [62]. Recent history of incarceration was associated with use of crystal methamphetamine among cis female sex workers in Iran [86].

#### Registration at a municipal health service

Four studies reported associations between mandatory registration at a city health service in Tijuana, Mexico and health outcomes [79,81,89,90]. One study suggested that registered sex workers had reduced odds of working in a sex work venue with high prevalence of HIV or syphilis and testing positive for HIV or an STI (syphilis, gonorrhoea, or chlamydia) univariably. These associations became insignificant after adjusting for injecting risk behaviours, age, and time in sex work [79]. Of note, sex workers who test positive for HIV in this system have their registration revoked, and sex workers already living with HIV cannot work in the regulated sector; therefore, sex workers who know or suspect they are living with HIV are unlikely to register. Registered sex workers had reduced odds of ever injecting drugs and higher odds of being tested for HIV [81]. A final study suggested that lack of registration was associated with increased odds of unprotected sex (OR 2.1, 95% CI 1.2–3.5, *p-*value not reported) [90].

#### Evaluation of sex work policies

Two studies in Canada evaluated a new policing guideline that prioritised enforcement of laws against clients and third parties over arrest of sex workers introduced in Vancouver in 2013. These studies found that there was no decrease in physical and sexual violence (OR 1.09, 95% CI 0.59–2.04, *p =* 0.78) among participants surveyed after 2013 compared to those surveyed before, but there was increased report of rushed negotiations with clients due to police presence (OR 1.73, 95% CI 1.03–2.90, *p-*value not reported) [57,92]. The introduction of an anti-trafficking policy in South Korea, accompanied by brothel closures, in 2010 was associated with a decrease in prevalence of gonorrhoea and antibodies to *Treponema pallidum* (indicating current or past infection), but also changes in the demographic profile of sex workers. Sex workers were younger in surveys conducted after the act compared to before, which may contribute to the lower prevalence of infection, although sex workers reported receiving more clients [77].

### Qualitative synthesis

#### Included qualitative studies

From the 94 eligible papers including qualitative data, we generated 4 core analytical categories over 37 unique analyses (papers) in different legislative frameworks and geographical settings, refining these through the inclusion of a further 9 purposively sampled papers (S3 Text). Studies were undertaken in a range of legislative models: Full criminalisation models were represented in 3 papers in the US; 2 papers each in Cambodia, Kenya, Serbia, South Africa, and Sri Lanka; and 1 paper each in Australia, China, Nepal, Pakistan, Uganda, and Zimbabwe. Partial criminalisation models were represented in analyses from 5 papers in Canada and 1 paper each in Hong Kong, India, Nigeria, Thailand, and the UK. Five papers focused on Canada following the introduction of criminalisation of clients, and 1 on Sweden, where that model is in place. Regulatory models—which criminalise those non-compliant with regulations including tolerance zones, regulated venues, and/or mandatory registration at a health care facility—were represented by 2 papers each from Australia, Guatemala, Mexico, and the US and 1 from Turkey. Four papers related to New Zealand, where sex work has been decriminalised. In total, interviews with 2,199 sex workers were analysed, representing a range of sex work locations (including street settings, truck stops, brothels, massage parlours, bars, night clubs, hotels, lodges, and homes) and means of meeting clients (including organised in person, via phone or online, independently, and via third parties). Most studies focused on cis women exclusively (*n =* 25), with a minority including sub-samples of trans women or transfeminine people (*n =* 18) or cis men (*n =* 9). Just 2 papers focused exclusively on the experiences of trans sex workers, and 1 on male sex workers. Ten studies included interviews with other actors associated with sex work, including clients, venue managers/owners, police, and outreach workers, but our analyses focused on data from sex workers themselves. Characteristics of included studies (data-rich and purposively sampled) [22,26,34–36,49,93–132] are summarised in Table 3, indicating which papers were purposively selected. A list of the other papers that were identified but not included is available (S3 Text).

**Table 3:** Summary of qualitative study characteristics included in the thematic analysis including legislative context and methods.

First author, year [reference]	Setting	Legislative model and policing*	Aim of study/article	Participants and recruitment	Methods	Focus of interviews/analysis
Abel, 2014^1^ [36]	New Zealand (various)	**Full decriminalisation.** All aspects of adult sex work decriminalised in 2003. Condom use required by law.	To present aspects of New Zealand’s experience with sex work decriminalisation, discussing process to get decriminalisation on policy agenda, way legislation was implemented, and impact on sex workers and wider community	58 sex workers (47 cis women, 9 trans people2, 2 cis men); aged 18–55 years. Ethnicities not reported. Main current sector: street, managed, private (most had worked in another sector in past). Recruited via sex worker organisation, by phone, and in sex work areas; maximum diversity sampling.	In-depth interviews and focus groups (within mixed-methods study). Thematic analysis. Members of sex worker organisation helped to develop interview guide and interpret data.	Impact of the Prostitution Reform Act, relationship with police and access to services.
Anderson, 2016 [93]	Vancouver, Canada	**Criminalisation of indoor venues and third parties.** In-call venues were subject to police raids, city inspections, licensing requirements, fines and license revocations, and enforced closures. National laws against operating a ‘bawdy house’ (i.e., sex work venue) and living off income generated via sex work were ruled unconstitutional during fieldwork.	Not stated, but the study is located within a community-based research project that aims to investigate the physical, social, and policy environments shaping sex workers’ sexual health, violence, HIV/STI risks, and access to care. Authors also stress the ‘need for research on the health and safety impact of sex work laws that criminalise managers and other third party actors who work in in-call sex work establishments’.	46 participants: 23 sex workers, 23 managers/owners (15 both workers and managers/owners). 45 cis women, 1 cis man (manager/owner). All migrants of Asian origin. Median age: 42 years (IQR 24–54). Recruited via outreach to in-call sex work venues and online.	Semi-structured interviews. Ethnographic observation (>430 hours) of physical and social aspects of indoor sex work environments. Thematic analysis (a priori and inductive). Research team included sex workers.	Experiences in the sex industry; interactions with police, city officials, co-workers, managers, and owners; and access to condoms, education, training, and outreach services.
Armstrong, 2014, 2015, 2016 [94–96]	Wellington and Christchurch, New Zealand	**Full decriminalisation.** All aspects of adult sex work decriminalised in 2003. Condom use required by law.	To examine how the decriminalisation of sex work impacts on violence risk management.	28 cis female sex workers, aged 17–57 years. Main current sector: street. 15 women identified as Maori (including 1 Cook Island Maori), 13 as New Zealand European. Recruited via sex worker organisations. 17 key informants working in agencies to support sex worker safety.	In-depth semi-structured interviews, observation. Analysis methods not described.	Entry into sex work, perceptions of risk, experiences of violence, strategies to manage risk, and impacts of the 2003 change in legislation.
Benoit, 2016 [97]	Canada (6 cities)	**Partial criminalisation.** Exchange of sexual services legal, but related activities illegal.^3^	Part of multi-project, community-engaged study examining perspectives and experience of 5 groups directly and indirectly affected by the sex industry. This paper focuses on sex workers’ perceptions and experiences with the police, to provide baseline data to assess the impact of legal change on sex workers’ confidence in police.	139 sex workers: 77% identified as women, 17% as men, 6% as other gender identities (including trans women and trans men). Mean age: 34 years (all 19 or older), 19% identified as indigenous, 12% as ‘visible minority’ (other ethnicities not reported). 22% worked on street, 54% indoors, and 24% in managed indoor work. Participants had to have right to work in Canada. Maximum diversity sampling.	Open-ended questions within structured interviews. Thematic analysis.	Interactions with police through sex work, perceptions of police attitudes, intersectional discrimination, and enhanced feelings of safety or danger.
Baratosy, 2017 [98]	Adelaide, Australia	**Partial criminalisation.** Criminalised activities include soliciting or loitering in public places; receiving money or being present in a brothel; and managing, keeping, or assisting to manage a brothel. In 2015 a decriminalisation bill was brought before parliament.	To explore the lived experiences of South Australian sex workers working within a criminalised setting to contribute evidence supporting decriminalisation in the South Australian context.	10 sex workers (7 cis women, 1 trans woman, 1 cis man, 1 gender-queer). Aged 31–68 years, working mostly off street (1 participant worked on street). Ethnicities not reported. Participants recruited via sex-worker-led peer support and education organisation.	Semi-structured interviews. Thematic, iterative analysis with reflections on researchers’ influence on interview. Sex worker involvement in study design.	Experience of sex work: police involvement, workplace protection, and health.
Biradavolu, 2009 [99]	Rajahmundry, India	**Partial criminalisation.** Act of selling sex not illegal, but promoting or profiting from sex work and all associated activities that make sex work possible are illegal.	To evaluate a community-led structural intervention for HIV prevention among sex workers (community mobilisations, changes in policing, establishment of community-based organisations).	75 cis female sex workers mostly working from home or street. Age and ethnicity not recorded. Participants recruited via outreach and through NGO. 11 interviews with NGO staff and 36 with lawyers, police, and other actors associated with sex work.	Interviews, observations of NGO meetings. Thematic analysis.	Involvement in intervention, law, policing, and policy environment of sex work in Rajahmundry, and life histories.
Brents, 2005 [100]	Nevada, US	**Regulation.** Licensed brothel system in counties with population < 400,000, with mandatory regular HIV and STI testing. Out-calls legal in certain counties, illegal in others. Illegal to live off earnings of sex work or coerce someone into sex work.	To examine the issue of violence within legalised brothels and analyse the mechanisms in brothels that address safety and inhibit risk of violence.	25 cis female sex workers recruited from 4 legalised brothels. Age and ethnicities not reported. 11 former brothel managers and owners, 10 activists, and 5 brothel customers also interviewed.	Semi-structured interviews, ethnographic observation of public debates. Thematic analysis.	Analysis focused on safety, violence, danger, risk, and fear.
Cepeda, 2014 [101]	Nuevo Laredo and Ciudad Juarez, Mexico	**Regulation.** Sex work legal in tolerance zones; registration, weekly HIV/STI testing, and valid health card mandatory. Illegal in all other areas.	To describe violence that sex workers experience and to understand the role of contextual constraints (e.g., venues, geographical context, gender system).	109 cis female sex workers, aged 18–46 years. All Mexican nationals (ethnicities not reported). Mapped then randomly selected locations/venues—included bars, clubs, hotels, dance bars, and street. Recruitment by outreach workers from local community.	Life history interviews. Grounded theory analysis (open then selective coding).	Demographics, career trajectory, clients, drug use, sexual behaviour, and HIV/AIDS.
Corriveau, 2014 [102]	Toronto, Ottawa, and Montreal, Canada	**Partial/quasi criminalisation.** Exchange of sexual services legal, but related activities illegal^3^; body rub parlours and low-barrier supportive housing unsanctioned.	To understand the experiences and views of adult male escorts of (1) criminal law relating to sex work and (2) strategies to cope with the legal situation.	19 cis male sex workers, all working as escorts, independently in clients’ homes or hotels; aged 19–41 years; majority (15) white Canadian, other ethnicities not reported. Recruitment via social and professional networks and flyers.	Semi-structured interview. Analytical methods not described.	Work experience and ambiguity of criminal law relating to sex work, and strategies used to cope with dangers of current legal climate.
Dewey, 2014 [103]	Denver, US	**Full criminalisation.** Selling and buying sex illegal. Location of first ‘end demand’ initiative in US in 1994—targeting clients of sex workers via intensified policing of street sex work locations.	To explore normative beliefs and practices that inform women’s decision-making processes as they interact with or seek to avoid police.	50 cis women working on the street, aged 18–63 years, majority African American, fewer identified as white, Latina, and Native American. Recruitment via snowball sampling.	Open-ended interview. Thematic analysis. Ethnographic approach (researcher lived in street sex work area to get to know participants).	How women define coercion in their everyday work experiences; women’s help-seeking practices and, within that, how they interact with police and social services.
Ediomo-Ubong, 2012 [104]^†^	Ikot Ekpene, Nigeria	**Partial criminalisation.** Criminalised activities include ownership or management of a brothel, underage sex work, and living off proceeds of sex work.	To understand experiences and decision-making in relation to drug use as a risk behaviour in life and work.	86 cis female sex workers working in brothels, identified through systematic sampling following mapping of all brothels in the area. Age and ethnicities not reported.	Focus groups and in-depth interviews. Textual and thematic analysis.	Drug use, factors motivating drug use, and effects on lives and work.
Foley, 2010 [105]	Dakar, Senegal	**Regulation.** Registered sex workers allowed to work legally (only cis women are eligible). Registration requires twice-monthly screening at STI clinic and presentation of health card; individuals’ details are sent to police. Public solicitation is illegal. Only 20% of sex workers are registered.	To identify key features of Senegal’s national HIV/AIDS policies and programmes.	60 registered and unregistered cis female sex workers, some of whom are living with HIV. All recruited via local NGO working with sex workers. Age and ethnicity not recorded. 10 government officials, physicians, NGO directors, and civil society leaders also interviewed.	4 community dialogue sessions with sex workers. Semi-structured interview guide for other participants. Content analysis.	Knowledge of HIV transmission, HIV/AIDS programmes, and ideas about vulnerability to HIV.
Ghimire, 2011 [106]^†^	Kathmandu Valley, Nepal	**De facto full criminalisation.** No legislation around sex work, but anti-trafficking laws used to regulate sex work and many policies used against sex workers.	To present individual, structural, and cultural factors facilitating or creating barriers to use of condoms among sex workers.	15 cis female sex workers, aged 19–42 years, purposively selected from a survey of 425 sex workers to represent diversity of ages, ethnicities, and marital and socio-economic statuses, working across a range of settings (restaurants, street, massage parlour). Majority were Janajati (ethnic minority group).	In depth interviews. Thematic analysis.	Knowledge and use of condoms, sexual activities and protective behaviour, potential partners, sexual harassment, and characteristics of partners.
Goldenberg, 2018 [132]	Tecún Umán, Guatemala	**Regulation.** Licensed indoor establishments with mandatory HIV/STI testing and health permits and informal street and indoor locations (hotels, motels, bars).	To examine the ways in which intersecting features of indoor work environments influence safety and agency to engage in HIV/STI prevention.	39 cis female migrant sex workers from Honduras, El Salvador, Nicaragua, Mexico, or Guatemala. Median age 27 years, working in formal venues with a health permit (27) and informal venues (17). Recruitment via community-based team of outreach workers with purposive sampling to ensure diverse range of migration experience.	Ethnographic: observations, focus groups, and in-depth interviews. Thematic analysis. Research guided by community advisory board of sex work, HIV, and women’s organisations.	Sex work and migration histories, working conditions, interactions with police and immigration and health authorities, violence, HIV/STIs, health service access, and other health concerns.
Gulcur, 2002 [107]^†^	Istanbul, Turkey	**Regulation.** Licensed brothels, with mandatory registration of sex workers including regular STI checks and ID cards. The systems is only for Turkish citizens.	To document the experience and working conditions of women who travel to Istanbul to undertake sex work.	3 cis female migrant sex workers from Eastern Europe and former Soviet Union countries (ages not reported) and 6 key informants (clients, sales people, and bartenders). Recruitment via hotels, bars, and businesses in district where sex work takes place.	Unstructured interviews. Thematic analysis.	Experiences and working conditions of migrant women as well as local discourses and attitudes surrounding migrant sex workers.
Ham, 2014 [108]	Melbourne, Australia	**Regulation.** Licensing framework for legal brothels and independent workers (Sex Work Act 1994), who are required to register and obtain licence. Medical certificate (STI screen) is required every 6 weeks.	To understand how sex workers’ agentic use of ‘strategic invisibility’ is affected by Melbourne’s sex work legalisation framework.	55 sex workers, mostly cis women (6 cis men, 2 trans women), working independently, as escorts, or in brothels. Majority white Australian, but 17 identified as South East Asian, English, Eastern European, or New Zealander. Participants recruited through fliers and email lists.	Open-ended interviews. Thematic analysis around key themes of stigma, health and well-being, and working conditions.	Working conditions.
Handlovsky, 2012 [109]^†^	Vancouver, Canada	**Partial/quasi criminalisation.** Exchange of sexual services legal, but related activities illegal^3^; body rub parlours and low-barrier supportive housing unsanctioned.	To investigate how condom use is practiced in massage parlours and as a social phenomenon situated within the nexus of supports and constraints.	21 individual and group interviews with cis female sex workers working in massage parlours. Mean age 30 years, 11 migrants from Asia. Recruitment via community outreach.	Conversational interviews. Thematic analysis. Sex workers involved as community researchers in linked survey (not reported if involved in qualitative component).	Condom use practices in commercial sex exchanges and personal, interpersonal, and structural level factors that influence use.
Huang, 2014 [110]^†^	China (6 cities and counties)	**Full criminalisation.** Criminalisation of purchase and sale of sex. Periodic crackdown on sex work with aim to eradicate sex work, as happened in 2010.	To explore strategies that female sex workers and managers adopted to deal with the 2010 police crackdown; discussion of the implications for health and HIV-related risks.	Interviews with 107 cis female sex workers. Ages and ethnicities not reported. 26 managers of sex work establishments, 13 outreach workers, and 24 health providers. Sex workers recruited through NGOs and sex work sites including hair salons, massage parlours, and street-based locations.	Observation and interviews. Thematic analysis.	Effects of police practices following the 2010 crackdown and strategies used in response.
Karim, 1995 [111]^†^	Truck stop mid-way between Durban and Johannesburg, South Africa	**Full criminalisation.** Criminalisation of purchase and sale of sex.	To explore the social context of risk of HIV infection.	Interviews with 10 cis female sex workers at truck stop, aged 17–34 years, all black (ethnicities not reported). Recruited via sex worker from setting trained in research methods. 9 interviews with truck drivers.	Interviews, field notes. Content analysis.	Social conditions at truck stop, sex work, family history, attitudes, and practices towards HIV/AIDS.
Katsulis, 2010 [35]	Tijuana, Mexico	**Regulation.** Sex work legal in tolerance zones; registration, weekly HIV/STI testing, and valid health card mandatory. Illegal in all other areas.	To examine the social context of workplace violence and risk avoidance in the context of legal regulation meant to reduce harms associated with sex work.	190 cis female sex workers recruited through STI clinics and in bars, clubs, and street settings, using snowball sampling following a mapping of sex work areas. Mean age 26 years, ethnicities not reported. Other interviews included police (4), hotel and bar owners (7), medical personnel (13), and community health outreach workers (23).	Ethnographic research included field observations and interviews. Grounded theory and thematic analysis.	Experience and management of violence at the hands of customers, strangers, and police.
Kiernan, 2016 [112]^†^	Goma, DRC	**Partial/quasi criminalisation.** Exchange of sexual services legal, but related activities illegal including forced sex work, but little government enforcement in reality.	To explore the experience of urban sex workers in eastern DRC in relation to violence, barriers to medical care, and use of local resources.	7 cis female and 1 cis male sex workers working in a night club, aged 23–34 years. Ethnicities not reported. Convenience sampling.	Semi-structured interviews. Thematic analysis.	Characteristics of sex work, exposure to violence, available resources, and access to medical care.
Krusi, 2012 [113]	British Columbia, Canada	**Partial/quasi criminalisation.** Exchange of sexual services legal, but related activities illegal^3^; body rub parlours and low-barrier supportive housing unsanctioned.	To report experiences of sex workers living and working in low-barrier supportive housing, focusing on how environments influence sex workers’ safety and risk negotiation with clients.	39 sex workers (38 cis women, 1 trans woman) living and working in low-barrier supportive housing. Aged 22–58 years (average 35), 30 of Aboriginal ancestry, 2 ‘other visible minorities’, 7 white. Recruited via 2 housing programmes.	In-depth interviews and focus groups. Content analysis. Focus groups co-facilitated by sex workers.	Experiences of living and working in low-barrier supportive housing, rules and regulations, police and staff relationships, safety, and negotiation.
Krusi, 2014 [114]	Vancouver, Canada	**De facto criminalisation of clients.** New police guidelines (2013) prioritised sex workers’ safety over enforcement, but continued to arrest clients.	To evaluate how enforcement against clients, but not sex workers, shapes sex workers’ interactions with police, negotiation of working conditions and transactions with clients, and protection against violence and HIV/STIs.	31 cis and trans female sex workers, aged 24–53 years. 8 of Aboriginal ancestry, 2 ‘other visible minorities’, 21 white. All had worked on street; now mainly sought clients on street (24) or by phone (7); provided services in vehicles/outdoors (27) or informal indoor venues (14). Purposive sampling via existing cohort study representing diversity in age, ethnicity, gender, and work environments.	Semi-structured interviews. Ethnographic observation of street sex work areas. Thematic analysis. Research and outreach team included sex workers.	Working conditions, interactions with police, and negotiations of health and safety with clients.
Krusi, 2016 [26]	Vancouver, Canada	**De facto criminalisation of clients.** New police guidelines (2013) prioritised sex workers’ safety over enforcement, but criminalised the purchase of sex, benefiting from the proceeds of sex work in an ‘exploitative’ fashion, advertising sexual services, and communication for the purpose of selling sexual services.	Part of a larger longitudinal qualitative and ethnographic study (AESHA) investigating how the physical, social, and policy environments shape working conditions and health of sex workers. This study aimed to explore the complex ways in which stigmatising assumptions of sex workers as ‘risky’ and ‘at risk’ intersect with evolving sex work policing strategies to shape street-based sex worker rights, experience of violence, and negotiation of sexual risk reduction.	31 sex workers (26 cis women, 5 trans women). Mean age 38 years; 8 of indigenous ancestry, 2 ‘other visible minorities’, 21 white. All had worked on street; now mainly sought clients on street (24) or by phone (7); provided services in vehicles/outdoors (27) or informal indoor venues (14). Purposive sampling via existing cohort study representing diversity in age, ethnicity, gender, and work environments.	Semi-structured interviews. Inductive and iterative thematic analysis, drawing on concepts of structural vulnerability, structural stigma, and everyday violence. Sex workers were involved in advising on the research.	Police interactions, working conditions, and negotiation of sex work transactions with clients after implementation of new policy.
Levy, 2014 [34]	Sweden (various)	**Criminalisation of clients.** In 1999, purchase of sex was criminalised and sale of sex decriminalised, but brothel-keeping charges remain.	Discusses the impact of Swedish sex purchase law on levels of sex work, sex work displacement, increasing dangers and difficulties of some types of sex work, service provision, and disruption of sex workers’ lives.	26 sex workers (22 cis women, 2 trans people^2^, 2 cis men); cis women working on street or as escorts, or stripping. Ages and ethnicities not reported. Also interviewed: clients, service providers, activists, police, and policy-makers. Recruited via public places, organisations attended by sex workers, and social networks.	Ethnographic participant observation and interviews. Grounded theory analysis. Co-author founded national sex worker rights organisation.	Not specified (see aim).
Lutnick, 2009 [115]	San Francisco, US	**Full criminalisation.** Selling and buying sex illegal. Proposal to decriminalise sex work, supported by Public Health Department and community groups, defeated in 2008.	To investigate the perspectives and experiences of a wide range of cis female sex workers regarding the legal status of sex work and the impact of the law on their working experiences.	40 cis women working in street and off-street settings. Average age 41 years; 18 African American, 16white, 3 Latin American, 2 Asian/Pacific Islander, and 1 Native American. Recruited through community-based organisations.	Semi-structured interview. Grounded theory analysis. Former and current sex workers involved in all aspects of study, including design, implementation, analysis, and write-up.	Social context of sex work, experiences with law enforcement, what work would be like if prostitution was not a criminal offence, and ideal legal framework for sex work.
Lyons, 2017 [116]	Canada, Vancouver	**De facto criminalisation of clients.** New police guidelines (2013) prioritised sex workers’ safety over enforcement, but continued to arrest clients.	To investigate the lived experience of violence and social-structural (social, political, and legal) contexts shaping violence among trans sex workers.	33 trans female sex workers, aged 23–52 years, 23 of indigenous origin, 7 white, 3 Filipino, Asian, or ‘other visible minority’. Majority worked on the street. Recruited via existing cohort.	In-depth interviews. Theory- and data-driven participatory analysis guided by ‘risk environment’ and ‘structural determinants’ framework. Sex workers were involved in the analysis.	Analysis focuses on how transphobia and criminalisation shape violence. Key themes: transphobia, clients’ discovery of gender identity, and negative police response to violence.
Maher, 2011 [117]	Phnom Penh, Cambodia	**De facto full criminalisation.** In 2008, trafficking law criminalised most aspects of sex work3; effectively made sale and purchase of sex illegal, led to police crackdowns and brothel closures.	To explore the relationship between sex work contexts and conditions and vulnerability to HIV/STI and related harms.	33 cis women aged 15–29 years working in brothels, entertainment venues, streets, and parks recruited through neighbourhood outreach by local NGO. Ethnicities not reported.	Inductive analysis drawing on principles of grounded theory.	Initiation into sex work, experience of sex work, conditions of sex work, drug and alcohol use, and culture and orientation towards prevention and use of HIV/STI services.
Maher, 2015 [118]	Phnom Penh, Cambodia	**De facto full criminalisation.** In 2008, trafficking law criminalised most aspects of sex work4; effectively made sale and purchase of sex illegal, led to police crackdowns and brothel closures.	To explore the impact of the 2008 trafficking law on sex workers’ HIV vulnerability and right to health.	80 interviews with cis female sex workers, aged 15–29 years, working in brothels, entertainment venues, streets, and parks. Ethnicities not reported. Recruited via community partner organisation (sampling methods not defined).	In-depth interviews. Iterative, inductive analysis guided by grounded theory.	Wave 1: impact of law and police crackdowns was a key emerging theme. Wave 2 (2011): impact of law on women’s lives.
Mayhew, 2009 [119]^†^	Rawalpindi and Abbottabad, Pakistan	**De facto full criminalisation.** Criminalisation of purchase and sale of sex, and third party making profits from sex work. Homosexuality illegal.	To investigate the nature and extent of human rights abuses against sex workers, transgender individuals, and people who inject drugs.	38 respondents (PWID, trans people, and sex workers) recruited through local NGO. Age and ethnicities not reported.	Participatory ethnographic and evaluation research, training peers to conduct interviews. Thematic analysis.	Complexities of gendered and sexual identities and nature and scale of abuse suffered.
Miller, 2002 [120]	Colombo, Sri Lanka	**De facto full criminalisation.** Criminalisation of purchase and sale of sex, and third party making profits from sex work. Lodges and massage clinics licensed, but sex work practiced covertly. Homosexuality illegal.	To investigate the routinization of violence and harassment against women and transgendered/gay men in an illegal sex market.	160 sex workers (107 cis women, 27 trans people, 26 cis men) recruited through snowball sampling and working across a range of settings (street, brothels, massage clinics). Age and ethnicities not reported. Also interviewed other people connected to sex industry (50) (e.g., managers, taxi drivers), clients (50), and criminal justice practitioners and NGO staff (15).	In-depth interviews. Thematic analysis around topic guide.	Relationship between cultural definitions of gender/sexuality and the implementation of existing legal frameworks, and impacts on treatment and experiences of sex workers.
Nichols, 2010 [49]	Colombo, Sri Lanka	**Full criminalisation.** Vagrants Ordinance penalises sex workers, third parties (and clients)^5^. Homosexuality illegal since colonial era; with rise in sex tourism, law increasingly targets male sex workers.	To examine how ‘gender and sexual orientation intersect to create unique configurations of abuses’ against transgender sex workers, compared with female sex workers.	24 interviews and 3 focus groups with transfeminine (‘nachichi’) sex workers, aged 18–42 years, working predominantly on street. Ethnicities not reported. Recruited by interviewers, via outreach to sex work settings and snowballing.	In-depth interviews and focus groups. Inductive, intersectional analysis: open then selective coding, categorising types of police abuse.	Background, education, employment, first sex, sex work, gender and sexual identity, and experiences with family, community, clients, and police regarding gender and sex work.
O’Doherty, 2011 [121]	Vancouver, Canada	**Partial/quasi criminalisation.** Exchange of sexual services legal, but related activities illegal^3^; body rub parlours and low-barrier supportive housing unsanctioned.	To share findings from research with off-street sex workers, focusing on their views of how criminal laws affect their work.	9 cis female sex workers, aged 22–44 years. None identified as Aboriginal or Métis (other ethnicities not reported). All independent; 8 had worked in other sectors in past (3 on street). Also interviewed 1 massage parlour owner/former sex worker. Recruited online (advertising on escort directory and secure website).	In-depth interviews. Analysis methods not reported. Former and current sex workers collaborated on the research.	Experiences of victimisation and work in indoor sex industry. Interviews identified common concerns and opinions about law.
Okal, 2011 [122]	Naivasha and Mombasa, Kenya	**Full criminalisation.** Many local authorities have specific bylaws against loitering or procuring for sex work or homosexuality. In Mombasa, consensual sex between men is criminalised. Often only sex workers, not clients, are taken to court for loitering or indecent exposure.	To examine the social and legal contexts that underpin the high levels of sexual and physical violence that pervade sex work in Kenya.	8 focus group discussions with 10–12 cis female sex workers aged 16–49 years, organised by natural groups, site of recruitment, and full/part-time sex work; recruited through HIV/AIDS peer educators and snowball sampling. Ethnicities not reported.	Focus group discussions. Content and thematic analysis.	Work, health, and contraceptive use.
Pitcher, 2014^2^ [123]	UK and Netherlands (various)	**Partial/quasi decriminalisation (UK).** **Regulation (Netherlands).** Sex work through licensed brothels legal for consenting adults, but illegal for individuals under 18 years old and migrants.	To compare the experiences of sex workers under different legal frameworks.	36 interviews with sex workers working in off-street venues, 2 managers, and 2 receptionists in massage parlours in UK (28 cis women, 9 cis men, 3 trans people). 30 identified as white UK, 6 as white European, 2 as white other, 2 as multiple ethnic groups.	In-depth interviews (UK only), comparative analysis of sex workers’ experiences under 2 different policies. Thematic analysis.	Experiences in sex work.
Pyett, 1999 [124]	Melbourne, Australia	**Regulation.** Legal in licensed brothels; illegal elsewhere (including escorting^6^/street). Condom use mandatory in licensed venues.	To explore issues of safe sex and risk management among sex workers who work on the street or in other criminalised sectors.	24 cis female sex workers, aged 14–47 years (average 28), working on street or in illegal brothels. Ethnicities not reported. Purposively sampled women perceived as potentially vulnerable.7	In-depth interviews. Content and thematic analysis. Sex workers involved in planning, recruitment, interviewing, and interpretation.	Managing work services, safety, stress, condom use, and relationships; worries, plans, health, caring, support, relaxation, disclosure, relationships, and child care problems.
Ratinthorn, 2009 [125]	Bangkok, Thailand	**Partial criminalisation.** Sex work allowed to operate in entertainment establishments, but street sex work is prosecuted under public nuisance and soliciting laws.	To explore characteristics of violence against sex workers and how violence influences personal and societal health risks.	28 cis women working on the street recruited via purposive, theoretical, and snowball sampling to select participants who had experienced violence. Recruited in work settings in 3 districts. Average age 32 years, all born in Thailand.	In-depth interviews, 1 focus group, observation of workplaces. Thematic analysis drawing on grounded theory techniques.	Presence and consequences of work-related violence; how violence threatened participants’ health, lives, and families; and their response to it.
Rocha-Jiménez, 2017 [126]	Tecún Umán and Quetzaltenango, Guatemala	**Regulation.** Change in legislation: sex workers no longer required to carry a registration card but must continue regular HIV/STI testing.	To explore how the implementation of public health practices (mandatory HIV/STI testing) shapes HIV prevention and care among migrant sex workers.	53 cis female sex workers, majority working in off-street venues. All participants Spanish-speaking with history of internal or cross-border migration. Average age 31 years. Recruitment via outreach and local NGO.	Focus groups and in-depth interviews. Thematic analysis. Research guided by community advisory board that included female sex workers.	Experiences with public health practices, related interactions with authorities (i.e., police), and HIV prevention and care.
Scorgie, 2013 [127]	Kenya, South Africa, Uganda, and Zimbabwe (various)	**Full criminalisation.** However, municipal bylaws and non-criminal legislation (e.g., loitering, public nuisance, indecent exposure) typically used to arrest and detain sex workers because easier to enforce.	To examine the combined effects of criminalisation and law enforcement on sex workers’ everyday lives and social relations and how they affect health and well-being.	Cis women (106), cis men (26), and trans women (4) working in a range of sex work settings (street, bar, hotel, and home) recruited through the African Sex Worker Alliance and snowball sampling. Mean age 25 to 35 years across sites, approximately 25% had history of internal or cross-border migration. Ethnicities not reported.	In-depth interviews and focus groups. Thematic analysis. Participatory approach: peer educators conducted interviews and checked analysis.	Experience of human rights violations by police, clients, regular partners, landlords, and others involved in the sex industry.
Shannon, 2008 [22]	Vancouver, Canada	**Partial criminalisation.** Purchase and sale of sex not illegal (at time of study), but laws against communicating and keeping a bawdy house (similar to soliciting and brothel-keeping laws, respectively).	To explore the role of social and structural violence and power relations in shaping the HIV risk environment and prevention practices of women in survival sex work.	46 women (cis and trans), average age 34 years, 57% identified as of Aboriginal origin. Recruited via purposive sampling following social mapping led by sex workers.	Focus groups. Thematic content analysis drawing on concepts of risk environment; structural, symbolic, and everyday violence; and relational notions of power. Participatory action research: survival sex workers involved in project conceptualization, implementation, and dissemination.	How sex work defined, relationships with clients and partners, descriptions/meanings of ‘bad date’ and safe environment, circumstances affecting power and control with clients, protective strategies, effectiveness of harm reduction services.
Sherman, 2015 [128]	Baltimore, US	**Full criminalisation.** Selling and buying sex illegal. In 2000–2007, intensified policing in low-income, minority neighbourhoods, including street sex work areas. Specialist prostitution squads can legally solicit/entrap sex workers.	To explore interactions between police and sex workers in professional and personal lives, in relation to broader HIV risk environment.	35 adult cis female sex workers; median age 37 years; 20 identified as African American, 15 as white. Purposive and snowball sampling. Recruited via organisations working with sex workers on street, in dance clubs, and in drug houses and via social network referrals.	In-depth interviews. Grounded theory analysis.	Entry into sex work, current work, condom use and negotiation, substance use, experiences of violence, and police interactions. Relevant themes: police repeatedly disregarding women’s safety, verbal and sexual harassment, and entrapment.
Rhodes, 2008, and Simic, 2009 [129,130]	Belgrade and Pancevo, Serbia	**Full criminalisation.** Criminalised under article 14 of the Law of Peace and Order.	To explore sex workers’ perception of HIV risk environment in Serbia.	24 cis women and 7 trans women working mostly in street sex work (beside busy roads, at railway and bus stations, at busy hotels) but some working via newspaper ads and in clubs/bars. Average age 28 years; 15 participants Roma (including all trans women, all working on the street), other ethnicities not reported. Recruitment via outreach services and snowballing.	Semi-structured interviews. Data collected in 2 waves to enable provisional coding and inform purposive sampling. Thematic analysis.	Entry into and modes of sex work, condom use and access, drug use, risk management, HIV and STI prevention, and health service need. Main themes: violence from police and clients, moral policing, and non-physical violence.
Wong, 2011 [131]^†^	Hong Kong	**Partial criminalisation.** Act of selling sex not illegal, but soliciting, keeping an establishment, or living on earnings of sex work is illegal.	To identify ways in which stigma may affect sex workers and how this links to health.	48 cis women selling sex working in a variety of venues (nightclubs, karaoke bars, brothels, and street) recruited through local NGO. Age not specified, 34 originated from Thailand, Philippines, Vietnam, or mainland China and 14 from Hong Kong.	In depth interviews. Data collection and analysis informed by grounded theory approach employing content analysis methods.	Experience and negotiation of sex-work-related stigma.

*Legislation and policing refers to at the time of the research.

^†^Papers purposively selected to reflect populations, settings, legislative models, and/or health issues under-reflected in the synthesis.

^1^For any methodological details not included in the paper, we retrieved this information from the original PhD thesis upon which the paper was based.

^2^Paper doesn’t specify whether trans women or trans men.

^3^Activities criminalised included communicating for prostitution in public spaces, procuring or living off the avails of prostitution, and keeping a bawdy house (i.e., brothel-keeping).

^4^Including public soliciting, procurement, managing a prostitution establishment, and providing premises for prostitution.

^5^Vagrants defined to include ‘those that engage in public loitering and prostitution’ including ‘aiding, abetting, or compelling a prostitute’.

^6^Escort agencies have since become eligible to register legally with the Prostitution Control Board, but were still criminalised during data collection.

^7^Considered vulnerable if young, inexperienced, homeless, drug or alcohol dependent, or working in illegal brothels or on the street.

DRC, Democratic Republic of the Congo; NGO, non-governmental organisation; PWID, people who inject drugs; STI, sexually transmitted infection.

Core analytical categories identified include disrupted workspaces and safety strategies; institutionalised violence, coercion, and extortion, and restricted access to justice; reproduction of multiple stigmas and inequalities; and restricted access to health and social care and support (S4 Text). Illustrative quotes from the core categories are summarised in Box 1.

#### Core category 1: Disrupted workspaces and safety strategies

In contexts of full or partial criminalisation, laws against soliciting or communication in public places for the purpose of prostitution—and feared or actual arrest—compromised street-based sex workers’ safety by rushing or displacing client screening and negotiations to secluded places, resulting in greater vulnerability to violence and theft by clients and others (Quote 1) [22,98,121,122,125,130]. For sex workers operating indoors, these laws impeded direct negotiations with clients and communication between peers about safety and sexual health [121]. This pattern persisted in contexts where clients were criminalised. Since it was in clients’ and sex workers’ mutual interest to avoid police detection, and because increased police presence and reduced number of clients led to the need to work longer hours [34,114], sex workers limited, rushed, or forewent usual client screening and negotiation, and were displaced to more isolated areas, increasing their exposure to violence and sexual health risks (Quotes 2, 3, 4a, and 4b) [34,114]. In Canada, cis and trans female sex workers continued to be displaced by police in areas undergoing gentrification, and, even when they were not targeted, some still experienced police presence as harassment [26,114]. Across diverse contexts, experience of possession of condoms being used as evidence of sex work, and experience of police raids where condoms had been confiscated, led to sex workers not carrying, using, or accessing condoms consistently [93,98,106,109] and venues restricting or not providing them [93,98,109,118]. In South Australia, sex workers attributed the latter to increased raids, closures, and the recent arrest of a venue owner [98].

Laws against brothel-keeping and bawdy houses left sex workers in the UK [123] and Canada [102,121] having to choose between working safely with other sex workers and/or third parties (e.g., security guards and drivers) and avoiding arrest by working in isolation (Quote 5), and deterred venue managers from providing sexual health training and supplies [93,121]. A lack of legal protection left sex workers vulnerable to exploitation by venue managers who could restrict access to information on their working and legal rights [121,123].

Anti-trafficking policies in Cambodia and attempts to ‘eliminate’ sex work in China resulted in police crackdowns on brothels, which displaced sex workers to unfamiliar and sometimes isolated locations (e.g., the street, bars, massage parlours, and private accommodations) where, working alone, they had less protection and control over negotiations with clients, lacked peer support to establish collective norms on condom use (Quote 6a and 6b), and were more vulnerable to sexual and other violence both from police and perpetrators posing as clients [110,117,118]. In Guatemala, some venue managers warned sex workers about raids, but, in common with experiences in Sri Lanka [120], others encouraged them to provide officers free sexual services to avoid their prosecution [132]. In India, some brothel owners paid police to avoid raids, or allowed pre-selected sex workers to be arrested [99]. Police harassment, raids [35,110,120], undercover operations, entrapment, and pressure to act as informants [97,128] generated fear, anxiety, and stress, with media sometimes publicising sex workers’ faces during raids [120].

Conversely, where certain indoor work places were informally approved by police in a wider landscape of criminalisation, as occurred in low-barrier housing for women in Canada, the removed threat of criminal penalties fostered venue-level safety strategies, in which sex workers could refuse unprotected sex or call the police in the event of a client becoming violent (Quote 7) [113]. Similarly, in the context of decriminalisation in New Zealand, cis female sex workers working on the street reported greater police presence contributing to their protection as well as increased time for screening clients (Quotes 8 and 9) [36,94–96]. Sex workers across sectors reported being able to negotiate services more directly and refuse clients [36]. Police became more focused on sharing information with women about violent incidents or individuals, and when their presence was off-putting to clients, women could request that they left [96]. Sex workers working outdoors no longer needed to move to isolated areas [94], although they continued to experience verbal and physical abuse by passers-by [95]. Although sex worker organisations objected to mandatory condom use within this model, some sex workers felt that it helped them insist on condom use [36].

In contexts of regulation in Australia, Mexico, and the US, venue-level systems such as alarms, fixed prices, intercoms, and condom use [100,124], as well as being able to work in close proximity with other sex workers and third parties [35,100,101,124], improved control and sense of safety for those able to work in regulated venues. Yet, in the US, some women criticised such systems as a veiled means of surveillance and as protecting management and clients’ interests above their own safety [100]. Across these settings, those unable to conceal venue-prohibited substance use were excluded from these premises and left as the authors note with ‘no choice but to work on the streets’ [124] or in the minority of venues where management overlooked these regulations [35,100,101]. In Canada, the cost of business licenses and the ineligibility of those with criminal records restricted access to and mobility between regulated venues [93,121]. In Mexico, only well-networked, resident, HIV-negative, cis female sex workers gained access to tolerance zones and regulated venues, which offered fewer physical risks than unregulated indoor and outdoor settings but were often overcrowded, making income less stable [35,101]. In Australia, Guatemala, and Mexico, the ineligibility of minors to work in regulated venues meant that they had to work on the street [35,124,126]. In Australia and Sri Lanka, sex workers operating in unregulated venues had less control over negotiations with clients, and some owners encouraged women to provide sex without a condom [124,120].

#### Core category 2: Institutionalised violence, coercion, and extortion, and restricted access to justice

Studies showed that policing practices in contexts of criminalisation and regulation institutionalised violence against sex workers, both directly through police inflicting physical or sexual violence or demanding fines in lieu of arrest, and indirectly by restricting access to justice and thus creating an environment of impunity for perpetrators of violence [97,102,122,125,127–130].

Violence and abuses of power by police were reported across all genders and diverse political and economic contexts, including Cambodia, Canada, the Democratic Republic of the Congo, India, Kenya, Nepal, Nigeria, Pakistan, Serbia, South Africa, Sri Lanka, Thailand, Uganda, the US, and Zimbabwe [49,97,99,104,106,111,112,118,119,122,125,127,128]. This took the form of arbitrary arrest and detention, verbal harassment, intimidation, humiliating and derogatory treatment, extortion, forcible displacement, physical violence, gang rape, and other forms of sexual violence during raids and in police custody [49,97,99,103,104,106,111,112,118,122,127,128]. In Kenya, Mexico, Nepal, Pakistan, Serbia, Sri Lanka, and the US, sex workers experienced extortion (unofficial ‘fines’, payments, or bribes) or provided sexual services enforced through physical or sexual violence or under threat of detention, arrest, transfer to rehabilitation centres, or forced registration (Quotes 10 and 11) [49,101,103,110,119,122,128–130], with limited or no opportunity to negotiate condom use [128]. Similar extortion and/or arbitrary fines were reported in China, India, Thailand, and Turkey (Quote 12) [99,107,110,125]. In Nepal, cis female sex workers, including those hired as peer educators, reported being arrested, beaten, and robbed by police upon being found in possession of condoms [106].

Reporting violence could result in sex workers’ being further criminalised [49,97,120–122,127,128]. Sex workers were reluctant to report violence and theft to the police [98,125] for fear of the following: arrest for prostitution-related activities, unrelated petty offences, or non-payment of previous fines [97,98,116,120,124,131]; being accused of crimes they had not committed [49,103]; harsh treatment or moral judgement [97,120]; further extortion or violence [35,101,112]; disclosure in court [97]; prohibitive costs [112]; or because no action would be taken to address the crime [97,111,112,114,116]. Long-standing discrimination, and the sense that police viewed them as criminals, made sex workers doubt the police would take complaints seriously [114,115,128]. When reports were submitted to police, sex workers’ accounts were dismissed as implausible, with police simultaneously blaming sex workers for the violence they had experienced [49,120,125], discrediting them as victims (Quote 13) [97,103,121,127,128], and sometimes further attacking or extorting them [49]. Cis and trans women in Canada and the US reported police questioning whether it is possible for a sex worker to be raped [97,128]. (Quote 14). Similarly, in Kenya, one cis woman reported being asked by an officer ‘how a prostitute like me could be raped as I was used to all sizes’, discouraging her from going to the police in future: ‘Never will I again go to report a case’ [127]. This produces an environment of impunity, where further violence, extortion, and theft from police and others operate unchecked [98,103,120,121,125,127], perceived to be a major contributor in normalising violence against sex workers [26,125].

Reluctance to report violence occurred even in contexts where the purchase but not the sale of sex was criminalised, due to fears that information about where sex work takes place could be used to target clients and harass sex workers (Quote 15) [34,114]. While some cis and trans women in Canada felt that police were now more concerned for their safety [26,114], others felt that officers continued to view them as ‘trash’, blame them for the violence they experienced, and deprioritise their safety [97], despite laws and police guidelines constructing them as victims [26]. In contexts of regulation, registered sex workers in Guatemala viewed their health cards (recording compliance with mandatory testing) as protective against police and immigration harassment [126,132], and registered sex workers in Mexico had better access to police protection but rarely reported violence [35]. In Senegal, registered workers still experienced being disbelieved when reporting physical or economic violence to police and so were reluctant to report it as a result (Quote 16) [105]. Concerns about being exposed to family and friends were paramount [35,105] and deterred some from registering [126]. Relationships with police were precarious, conditional on maintaining registered status, which can vary each month depending on compliance with mandatory screening requirements—with those whose registration has (temporarily) lapsed facing arrest, detention, and/or fines (Quote 17) [35,126]. Those who were not registered were afraid they would be sent to jail or fined for working illegally, or for active drug use [35], and were more heavily targeted by police for fines, arrest, detention, extortion, and sometimes sexual violence [35,101,124]. In India, marked reductions in police raids and violence were achieved through a peer-based intervention that facilitated access to justice and challenged power relations between sex workers and police, although some officers cited lengthy procedures to dissuade reporting [99]. In Canada, Mexico, Thailand, and the US, some sex workers described certain officers’ concern for their safety and support, but such concern was the exception [35,97,103,125].

Since decriminalisation in New Zealand, sex workers describe having better relationships with the police, and greater access to justice which—despite some prevailing mistrust in police—makes them feel safer and more confident with clients [36,95,96] and more deserving of respect (Quote 18) [36]. The removal of threat of arrest—which reduced police power and afforded sex workers rights—gave sex workers, and particularly young people [95], greater confidence to report violent incidents, exploitation by managers, and disputes with clients [36,96]. However, some officers treated disputes with clients as breaches of contract rather than crimes [96]. While there were still some reports of abuses of police power, there were also examples of offending officers being prosecuted as a result, helping to challenge environments of impunity [36,94,96].

#### Core category 3: Reproduction of multiple stigmas and inequalities

Findings show that repressive police treatment reinforced inequalities and entrenched marginalisation of sex workers, as well as creating disparities within sex-working communities, with police targeting specific settings or populations. In the context of full criminalisation in Sri Lanka, sex workers reported experiencing harsher punishment than their clients or managers: both sex workers and clients might be fined, but clients were not arrested or charged in the way that sex workers were [49], nor were managers of flats arrested during police raids [120]. Across settings, arrests, fines, extortion, and theft by police particularly targeted street-based sex workers [101,103,120,128], resulting in loss of income and increased economic vulnerabilities (Quote 19) [49,99,103,118,125,127,129,130]. Findings from Canada, Sri Lanka, and the US also show how criminalisation and police enforcement restricted freedom of movement, as sex workers were targeted arbitrarily by police during and outside of sex work hours and environments [49,97,103,120,128], and outed as sex workers by officers [97].

Studies showed how police targeting and mistreatment of sex workers, and inaccessibility to justice, reproduced inequalities and discrimination against sexual and gender minorities [26,49,116,119,127,129,130], people who use drugs [22,103,128,133], women, people of colour, and migrants [26,34,97,98,128,129,132]. In Serbia, Roma trans sex workers were treated with ‘contempt’ both by police enacting ‘extreme violence’ against them and by clients who expected cis women (Quote 20) [129]. In sub-Saharan Africa, male and trans sex workers described the ‘double stigma’ they faced, which could result in humiliation, ostracisation, eviction, and lack of access to micro-finance schemes, and this was worse in settings where homosexuality is also criminalised (Quote 21) [127]. In Sri Lanka, where both sex work and homosexuality are criminalised, trans sex workers were less likely to be charged than cis women but they experienced extensive extortion, humiliation, false accusations of crime, and verbal, physical, and sexual violence by officers targeting their gender expression (Quote 22) [49,120]. Similar experiences were reported among feminine-presenting male and trans sex workers in Pakistan and among trans women and sex workers of colour in Canada and the US [26,119,128]. In Canada, trans sex workers attributed officers’ lack of response to their reports of violence to the stigma and discrimination surrounding their gender, sex work, and drug use, reinforcing their self-blame [116].

Long-standing racial discrimination and community mistrust reinforced black and indigenous sex workers’ doubts that the police would take their complaints of violence seriously [26,128], and drug use was used to undermine sex workers’ testimony against their attackers (Quote 23) [128]. In the US, one woman described what police said to an ex-boyfriend who had beaten her up: ‘You can’t go hitting her, even though I’d hit her for being a junkie’ [128]. In Canada, a cis female independent sex worker described a police officer calling her ‘just a fat…native whore’ [97], while some white male independent sex workers attributed their lack of police attention to their race and social and economic privilege [102].

In criminalised and regulated settings, the precarious legal status of undocumented or unregistered migrant sex workers was used by clients [127] and venue owners [132] to refuse payment, and by landlords to charge inflated rents for substandard rooms [107]. Migrant sex workers did not report violence and other crimes to the police due to fear of deportation [35,131,132] or language barriers [98]. In Guatemala, police officers sometimes rounded up migrant sex workers whether or not they were registered [126], and in Turkey, police targeted ‘foreign-looking’ women presumed to be migrant sex workers [107]. In Sweden, immigration legislation and anti-trafficking policies have been used to deport migrant sex workers, despite their characterisation in national prostitution law as victims of violence, as a way of reducing sex work [34].

#### Core category 4: Restricted access to health and social care and support

Research demonstrates how criminalisation and police enforcement restrict sex workers’ access to health and social care. In Cambodia and various sub-Saharan African countries, crackdowns on brothels have reduced access to health services by disrupting peer networks and displacing sex workers from usual places of work, making it difficult for outreach services to find people, and hindering collective organisation (Quote 24) [118,127]. In China, sex workers were reluctant to accept condoms from health services after police crackdowns, for fear of their use as evidence [110]. In Sweden, the mandate to reduce sex work acted as a barrier to services, as sex workers’ access became conditional on leaving the sex trade and conforming to a victim discourse, and health services no longer distributed condoms through outreach [34]. Based on ethnographic observations, authors noted multiple difficulties experienced by sex workers as a result of laws against renting property used for sex work, including problems with eviction as well as with immigration, child custody, and tax authorities [34]. In Canada, some sex workers had received referrals from supportive police to health, counselling, and legal aid services [97], but indoor venue managers remained reluctant to allow outreach visits for fear of prosecution, restricting access to sexual and broader healthcare—particularly disadvantaging migrant sex workers who relied on outreach [93]. Trans sex workers in Canada [116] and sex workers of all genders in South Australia [98] were fearful of accessing clinics [116], sex-worker-led outreach services, and peer information and resources [98], for fear of being reported to the police.

Studies showed how registration and mandatory testing necessitated more frequent contact with healthcare systems [100,108,115,132] and were viewed positively by authors in Nevada, US, as a way of maintaining a low level of STIs [100] and by some sex workers as a form of self-responsibility for health [108,126]. However, in Guatemala the decision to comply with testing requirements was mostly motivated by fear of police harassment and detention rather than health considerations [126,132]. In Turkey, unregistered migrant sex workers were forcibly tested upon arrest [107], and in Australia, some sex workers experienced judgement and were refused testing by health professionals [108]. Mandatory testing of sex workers is considered a rights violation by the UN Refugee Agency and the Joint United Nations Programme on HIV/AIDS that can create barriers to sex workers accessing voluntary services and can facilitate discrimination against sex workers living with HIV. In Nevada, sex workers who test HIV positive can face up to 10 years in prison if they are found selling sex in a licensed or an unlicensed environment [100]. Discrimination against sex workers in general was often reinforced, and mandatory registration was not only time-consuming but could lead to public disclosure of sex work, adversely affecting individuals’ credit rating and ability to obtain a loan (Quotes 25–28) [108,115,127]. Regulation systems also restricted migrants’ access to sexual health services [35], and those with undocumented status in Turkey lacked broader access to healthcare and banking services, leaving them vulnerable to theft [107]. In Canada, sex workers’ fear of becoming known to the authorities left them dependent on cash and unable to access loans [107,121].

**Box 1. Quotes**

**Core category 1: Disrupted work spaces and safety strategies**

Quote 1: ‘They couldn’t have designed a law better to make it less safe, even if they sat for years! It’s like you have to hide out, you can’t talk to a guy, and there’s no discussion about what you’re willing to do and for how much. The negotiation has to take place afterwards, which is always so much scarier. And you’re in a parking lot somewhere with some dude and all of a sudden he decides he doesn’t want to pay that, or pay anything at all and what are you going to do about it? So, yeah, it’s designed to set it up to be dangerous. I don’t think it was the original intention, but that’s what it does.’—cis woman, sector and age unspecified, Canada [121]

Quote 2: ‘Twenty seconds, one minute, two minutes, you have to decide if you should go into this person’s car…now I guess if I’m standing there, and the guy, he will be really scared to pick me up, and he will wave with his hand “Come here, we can go here round the corner, and make up the arrangement”, and that would be much more dangerous.’—cis woman, internet escort/street, age unspecified, Canada [34]

Quote 3: ‘While they’re going around chasing johns away from pulling up beside you, I have to stay out for longer.…Whereas if we weren’t harassed we would be able to be more choosy as to where we get in, who we get in with you know what I mean? Because of being so cold and being harassed I got into a car where I normally wouldn’t have. The guy didn’t look at my face right away. And I just hopped in cause I was cold and tired of standing out there. And you know, he put something to my throat. And I had to do it for nothing. Whereas I woulda made sure he looked at me, if I hadn’t been waiting out there so long.’—cis woman, street, age unspecified, Canada [114]

Quote 4a: ‘Sometimes the guy will drive up and just sort of wave or point to go down the alley or something like that somewhere else where he can pick me up. [How does that affect your safety?] You never know who it is, right? And you can’t really see his face, can’t really see anything they could have a gun in their hand or. You know what I mean they could be a little drunk or something if you can’t really see them very clearly, you know. And you don’t you can’t say hi or whatever before you get in. You have to just hurry up before the cops come.’—cis woman, street, age unspecified, Canada [114]

Quote 4b: ‘Clients are worried about police. To avoid police they wanna move to a different area. I don’t want to go out of my zone right.…Once you get out there, like you know their turf so it’s harder for me cause it’s their comfort zone so they act differently, you know what I mean. Yeah it never ends up good’—cis woman, street, age unspecified, Canada [114]

Quote 5: ‘The ideal situation is where you…have a separate premises where you can work from, and share those premises…Because then you’ve got companionship, added security, there’s someone to interact with. Because of the legal situation you have to be very, very careful. Because obviously it’s running a brothel, which has…really dangerous consequences these days.’—cis man, independent, age unspecified, UK [123]

Quote 6a: ‘In the past, we just stay in the brothel and no one dared to hurt us or beat us because we are there in the brothel. But now [since police crackdowns] we cannot know where they take us to. Such as taking us to Prek Ho [a village 15 km from Phnom Penh] and hurt us. We don’t know in advance. There is no one to control us. So it is not safe for us.’—cis woman, formerly brothel-based, age 26 years, Cambodia [118]

Quote 6b: ‘Now some clients may force us not to use condoms but when we lived in the brothel we had more rights than clients and they dared not to force us because they come into our house.’—cis woman, formerly brothel-based, age 22 years, Cambodia [118]

Quote 7: ‘One of the staff caught one [a violent client]. He was a visitor in the house, and he came in as a date, and they called the police, and he got arrested.’—cis woman, indoor, age unspecified, Canada [113]

Quote 8: ‘And the police weren’t around as much (before decriminalization). But when it got legalised the police were everywhere. We always have police coming up and down the street every night, and we’d even have them coming over to make sure that we were all right and making sure our minders, that we’ve got minders and that they were taking registration plates and the identity of the clients. So it was, it changed the whole street, it’s changed everything.’—cis woman, street, age unspecified, New Zealand [36]

Quote 9: ‘You stand outside the car and talk. Don’t get in the car and talk—it’s best to just get them to wind the window down, stand there, talk to them and judge them. Yeah.’—cis woman, street, age unspecified, New Zealand [94]

**Core category 2: Institutionalised violence, coercion, and extortion, and restricted access to justice**

Quote 10: ‘There was this time when I was arrested by six policemen. They afterwards demanded sex from me. One of them threatened to stab me if I refused. I ended up having sex with all of them and the experience was so painful.’—cis man, sector unspecified, age 26 years, Kenya [127]

Quote 11: ‘It’s really pathetic taking money from us. I don’t know how they don’t understand I struggled for that. I sold my body. I worked. The man, for instance, pardon me, fucked me and everything, for the money. And they take the money. Why? I don’t know, but so they say it goes into some fund, what do I know?’—cis woman, street, age not specified, Serbia [129]

Quote 12: ‘Does the law limit how much they [police] charge [when fining sex workers]? Today, 500, tomorrow 300. Why the law does not limit…the charges for this amount? For gambling, 1000 charged, prostitution 500, isn’t there a limit? We don’t understand. I feel like the charges just depend on their [police] mood.’—cis woman, focus group, sector and age not specified, Thailand [125]

Quote 13: [In a case where a participant reported being attacked by a client and the case going to court.] ‘He ended up getting off even though I had photos of the bruises. This is likely related to the institutional attitude that women who sell sex deserve what they get from taking on a dangerous occupation—it’s such bullshit but so common! Also, I feared prosecution myself as a prostitute so I was unable to be completely truthful in court and my abuser was let off—even with the evidence’—cis woman, independent off street, age not specified, Canada [121]

Quote 14: ‘The police don’t look at us as victims when we’re raped and when we’re beaten and stuff like that. If we get into a physical altercation and we have to fight for our lives, we’re most likely to be jailed because of it.’—cis woman, sector not specified, age 40 years, US [128]

Quote 15: ‘They come to my door and, you know, ask for my ID and so forth so it’s like harassment…The third time it’s like, “We know what you’re doing, I mean, what you’re about. We’re going to go after your clients”…I make a living out of this, so I was really paranoid for a very long time after.’—cis woman, internet escort, age not specified, Sweden [34]

Quote 16: ‘One night a client went off with a girl, and after their encounter he beat her. The next day she recognised him in the bar and told the bar owner who told her to go to the police. When she got to the police station the officers didn’t believe her—they said she didn’t have any proof. The police don’t give us any help at all.’—cis women, working in a bar with registration, age not specified, Senegal [105]

Quote 17: ‘Once, I forgot to return [to the city clinic] for a health stamp. The police threatened to take me and nine other girls to jail, but they let us go with a warning and a 2,000 pesos fine [$220].’—cis woman, sector not specified, age 19 years, Mexico [35]

Quote 18: ‘Well it definitely makes me feel like, if anything were to go wrong, then it’s much more easier for me to get my voice heard. And I also, I also feel like it’s some kind of hope that there’s slowly going to be more tolerance perhaps of you know, what it is to be a sex worker. And it affects my work, I think…when I’m in a room with a client…I feel like I am deserving of more respect because I’m not doing something that’s illegal. So I guess it gives me a lot more confidence with a client because, you know, I’m doing something that’s legal, and there’s no way that they can, you know, dispute that. And you know, I feel like if I’m in a room with a client, then it’s safer, because, you know, maybe if it wasn’t legal, then, you know, he could use that against me or threaten me with something, or you know. But now that it’s legal, they can’t do that.’—cis woman, sector and age not specified, New Zealand [36]

**Core category 3: Reproduction of multiple stigmas and inequalities**

Quote 19: ‘Now if I get caught to police people, they check pockets and all and take everything.…the police people will snatch it [money] away…Even if we find two hundred [rupees] a police person will come [and take it].’—trans woman (nachichi), street, age unspecified, Sri Lanka [49]

Quote 20: ‘They [police] started going wild, only on us transvestites. They let the girls go. They just pick us up, and go to the woods, and go wild on us…First, they beat us in the woods, and then they take us to the station. And then they tell us at the station “Hey, freshen up,” and they beat us up in the bathroom’—transvestite [author’s term], street, age unspecified, Serbia [129]

Quote 21: ‘Sometimes a man will take you and after fucking, he says, “You are gay, where can you report me? I’m not paying you and you can do nothing about it.”‘—cis man, focus group, sector and age unspecified, Uganda [127]

Quote 22: [After reporting being jailed on charges of prostitution and describing an incident with police involving forced gender behaviour] ‘I’m very scared of policemen of course.…They straight away tell.…“Go sing a song! sweep!” Talk to us like dogs.’—trans woman, street, age unspecified, Sri Lanka [49]

Quote 23: ‘Because it wasn’t a trial of rape, it was a trial of me being a heroin addict, me being on methadone. It got thrown out of court….’—cis woman, street, age unspecified, Canada [22]

**Core category 4: Restricted access to health and social care and support**

Quote 24: ‘Since the new law was passed, fewer women access health care and prevention services because we live at different places nowadays and NGOs could not find us. In the past, women live in one place at the brothel. We also want to contact NGOs but we don’t know the location of the NGOs…So we could not access to prevention services…Since the brothel was closed I have never contacted it again.’—cis woman, brothel, age 22 years, Cambodia [118]

Quote 25: ‘Because the policemen crack down often we cannot earn money. We are sleepless, so we sleep at day time, so I am lazy to go to check my health. I have no feeling to go.’—cis woman, brothel, age 22 years, Cambodia [118]

Quote 26: ‘I think every month is stupid. It has to be every three months at least. Because it’s a pain for owners, it’s a pain for girls, for everyone, because like you can’t go to your family doctor and say, “Listen I need a certificate”. You have to go to a sexual health clinic and wait all day to see a doctor.’—cis woman, brothel and escort, age unspecified, Australia [108]

Quote 27: ‘[For] any insurance one of the questions is, “Have you been a prostitute?” Whatever, now if they pulled your health records and they saw how many tests you’d had, you can’t lie about that one and I think it should be totally illegal [insurance companies asking about sex work]. And I would like to see them do a bit of a study on girls in the sex industry who have worked, that aren’t on drugs and how many diseases they actually have, to see if this kind of discrimination is warranted, because it’s not.’—cis woman, sector and age unspecified, US [108]

Quote 28: ‘I worked in a legal prostitution setting in Nevada. I did that for a couple of weeks to see what it was like. The amount of controls and the lack of freedom was horrendous. You know, I don’t want someone else telling me how to work. And I don’t think it is necessary really. Yeah, I think decriminalization gives us the most freedom.’—cis woman, independent in-call and out-call, age 39 years, US [115]

## Discussion

We estimate that, collectively, lawful or unlawful repressive policing practices linked to sex work criminalisation (partial or full) are associated with increased risk of infection with HIV or STIs, sexual or physical violence from clients or intimate partners, and condomless sex. The qualitative synthesis clearly shows pathways through which these policing practices and health risks are associated: enacted or feared police enforcement—targeting sex workers, clients, or third parties organising sex work—displaces sex workers into isolated and dangerous work locations and disrupts risk reduction strategies, such as screening and negotiating with clients, carrying condoms, and working with others. Specific policing practices, including confiscation of condoms or needles/syringes, are associated with increased odds of HIV, STIs, and violence by a range of actors. Repressive police practices frequently constitute basic violations of human rights, including unlawful arrest and detention, extortion, physical and sexual violence by law enforcement, lack of recourse to justice, and forced HIV testing—violations inextricably linked to increased unprotected sex, transmission of HIV and STIs, increased violence from all actors, and poorer access to health services [3,29,134]. The qualitative synthesis shows how violence and stigma against sex workers are institutionalised, legitimised, and rendered invisible [26,35] in contexts of any criminalisation and regulation [26,35], as sex workers across settings consistently report being further criminalised, blamed, or ignored when they report crimes against them. This structural, symbolic, and everyday violence fosters climates of impunity and under-reporting, and failure to recognise sex workers as citizens deserving protection, care, and support [26]. Targeting and exclusion of the most marginalised sex workers reinforces and obscures the injustices they face.

Our findings build on previous reviews documenting the extent to which and how social and structural factors influence sex workers’ safety and vulnerability to HIV. They do so by showing how these factors interplay with criminalisation to further marginalise sex workers and deprive them of civil, labour, and social rights [134–137]. Fear of prosecution and moral judgement, due to laws against homosexuality and transgenderism [138] and drug use [135], and, in the case of migrant workers [139], fear of deportation, further reduce willingness to report violence and exploitation to the police. Other evidence has shown how evictions based on landlords’ fears of brothel-keeping charges increase vulnerability to homelessness for sex workers and their families, while arrest and criminal records or simply being identified as a sex worker can lead to sex workers’ children being placed in institutional care [135,140].

Despite including search terms relating to broader health outcomes, the majority of epidemiological literature focused on sexual health outcomes and, in more recent evidence, violence. We found few studies that focused on emotional health, but these show detrimental associations with repressive policing and criminalisation. Qualitative and quantitative studies demonstrate that police enforcement and its threat is a major source of anxiety [103,141], whereas working in indoor, decriminalised environments is associated with improved mental health outcomes [32,142]. A recent critical literature review demonstrates that criminalisation, stigma, poor working conditions, isolation from peer and social networks, and financial insecurity have negative repercussions for sex workers’ mental health [13]. Only 1 quantitative study reported on the associations between policing and violence from intimate or other partners, and further research is needed to understand the mechanisms of this relationship [58]. It is clear that criminalisation and stigma interact to reproduce sex workers’ exposure to physical and sexual violence, and limit possibilities to resist or challenge it, and interventions are urgently needed to address violence against sex workers from all perpetrators. Successful sex-worker-led approaches to improving access to justice and challenging institutional stigma in South India offer important examples of what can be achieved with sustained funding and support [99].

Findings clearly show that criminally enforced regulatory models create major disparities within sex worker communities, possibly enabling access to safer conditions for some but excluding the large majority who remain under a system of criminalisation, including trans women, cis men, people who use drugs, migrant populations, and often sex workers operating in outdoor environments, who are at increased risk of HIV in many settings [81,90,126]. In contexts of mandatory HIV testing following arrest, fear of enforcement can hinder voluntary uptake of HIV testing and interventions [71,80], showing how this punitive approach to public health ultimately reduces access to health services. More recent research from Senegal has shown that while registration was associated with better physical health, the stigma attached to being registered has a detrimental effect on well-being; only a minority of sex workers are registered, and those who test HIV positive are excluded [143]. As the qualitative synthesis demonstrates, in New Zealand, following decriminalisation, sex workers reported being better able to refuse clients and insist on condom use, amid improved relationships with police and managers [36,144,145]. Other research in this setting indicates that decriminalisation has the potential not only to reduce discrimination, denials of justice, denigration, and verbal abuse but also to improve sex workers’ emotional well-being [31]. This concords with existing modelling data that suggest a positive effect of decriminalisation on incidence of HIV [2].

We were unable to examine the effects of different legislative models in the quantitative synthesis due to limited data, particularly for the models of decriminalisation and the criminalisation of the purchase of sex. Evidence included in our qualitative synthesis clearly shows that criminalisation of clients does not facilitate access to services, nor minimise violence. This is supported by the epidemiological evidence from Vancouver that showed that sex workers who were stopped, searched, or arrested were at increased risk of client violence despite the introduction of more severe laws against the purchase of sex introduced in 2014 (alongside fewer sanctions for sex workers working together and modelled on the Swedish law) [57]. In addition, the practice of rushing negotiations due to police presence increased and was associated with increased client-perpetrated violence [92]. Findings from our qualitative synthesis suggest that enforcement strategies that seek to reduce the numbers of sex workers [118] or clients [114] are unlikely to achieve these effects, since the economic needs of sex workers remain unchanged, resulting in sex workers having to work longer hours, accept greater risks, and deprioritise health. There is no reliable evidence from Sweden that the numbers of sex workers have decreased since the law changed in 1999 [34].

### Limitations

There are a number of limitations to this review. Findings from our pooled meta-analyses examining condom use and violence were limited by high heterogeneity, although effect estimates remained consistent across sensitivity analyses, suggesting we can be confident in their robustness. By limiting the search to literature written in English, Russian, and Spanish, we may have missed key studies. There was a lack of comparable quantitative data on outcomes such as access to services, drug-related harms, and emotional ill health, which precluded the use of meta-analysis. Similarly, few qualitative studies explored the emotional health effects of criminalisation and enforcement, and its effects on access to health and broader services received less attention relative to safety and health risks, within the rich body of evidence reviewed. Methodologically, some studies did not provide sufficient detail on sampling and analysis methods, and few included reflexive discussions on the position of the researcher. Although a growing number involve sex workers as researchers or advisors, few included discussion of the challenges and benefits of participatory approaches. We found few eligible studies that included trans female or cis male sex workers, who experience particular inequalities in relation to HIV, access to services, and—as the qualitative synthesis shows—police targeting and violence, limiting our ability to generalise findings to these populations. It is also possible that some studies may not have differentiated between trans women and cis men [146], or between cis and trans participants within samples of female and male sex workers, and few disaggregated experiences or outcomes by gender. This is an important area of future research given the specific vulnerabilities experienced by these populations, in contexts where gender and sexual minorities are criminalised, inadequately protected against hate crimes, and, in the case of trans people, not legally recognised. There is particular need for research with trans women, who experience intense violence, discrimination, and exclusion from education and employment, and whose health needs have been obscured by their conflation with ‘men who have sex with men’ [146].

Our review focuses on the implementation of enforcement practices linked to 5 broad legislative models. While it is clear that sex work laws and enforcement practices are inextricably integrated and it is key to link practice to legal frameworks to inform policy-making and advocacy, our findings reinforce previous evidence [37,38] that shows wide variation in how laws are enforced, which vary with sex work setting [126], visibility of sex work, sex workers’ and managers’ relationships with individual officers [99,101], and political and media attention [110,125], or arbitrarily by city [121]. We report on recent and past history of arrest or prison based on the information available to us, but few studies reported whether the arrest was related to sex work, was related to another offence, or had to do with social, gender, or racial profiling. Assessing the extent to which the enforcement practice was lawful or unlawful is beyond the scope of this review, but in some cases unlawful activities are clearly evidenced (e.g., police violence) while in others they are less visible or evidenced. This limits our ability to assess the specific contribution of sex work penalties to the health and safety of sex workers, relative to the use of other penalties and abuses of police powers against sex workers in contexts of criminalisation. Lack of clarity on the lawfulness of police enforcement practices also reflects the difficulties in measuring stigma and its interaction with criminalisation, and the need for mixed-methods approaches to unpack these complexities in context. We found few data on the interplay between criminalisation, collective organisation, and health outcomes. Evidence from India has shown how tackling social injustice and mistreatment by the police as part of a sex-worker-led HIV prevention intervention has resulted in fewer arrests, more explanation of reasons for arrest, and fairer treatment by the police, as well as decreased violence against sex workers [84,99]. However, most evaluations of community-led health interventions have been limited to HIV prevention and have been implemented in India, Dominican Republic, and Brazil [147,148]. Although there are numerous examples of active sex worker organisations advocating for sex worker rights and evidence-based policy internationally, as well as developing guidelines for rights-based HIV programming with, for, and by sex workers [149], the voices of sex workers continue to be dismissed and silenced in policy debates in many settings as well as in the design and evaluation of public health interventions.

## Conclusion

The public health evidence clearly shows the harms associated with all forms of sex work criminalisation, including regulatory systems, which effectively leave the most marginalised, and typically the majority of, sex workers outside of the law. These legislative models deprioritise sex workers’ safety, health, and rights and hinder access to due process of law. The evidence available suggests that decriminalisation can improve relationships between sex workers and the police, increasing ability to report incidences of violence and facilitate access to services [36,95,96]. Considering these findings within a human rights framework, they highlight the urgency of reforming policies and laws shown to increase health harms and act as barriers to the realisation of health, removing laws and enforcement against sex workers and clients, and building in health and safety protections [134]. It is clear that while legislative change is key, it is not enough on its own. Law reform needs to be accompanied by policies and political commitment to reducing structural inequalities, stigma, and exclusion—including introducing anti-discrimination and hate crime laws that protect sex workers and sexual, gender, racial, and ethnic minorities. Mixed-methods, interdisciplinary, and participatory research is needed to document the context-specific ways in which criminalisation or decriminalisation interacts with other structural factors and policies related to stigma, poverty, migration, housing, and sex worker collective organising, to inform locally relevant interventions alongside legal reform. This research must go alongside efforts to examine concerns surrounding decriminalisation of sex work within institutions and communities, which influence policy and practice, and sex workers must be involved in decision-making over any such research and reforms [121,150]. Opponents of decriminalisation of sex work often voice concerns that decriminalisation normalises violence and gender inequalities, but what is clear from our review is that criminalisation does just this by restricting sex workers’ access to justice and reinforcing the marginalisation of already-marginalised women and sexual and gender minorities. The recognition of sex work as an occupation is an important step towards conferring social, labour, and civil rights on all sex workers, and this must be accompanied by concerted efforts to challenge and redress cultures of discrimination and violence against people who sell sex. While such reforms and related institutional shifts are likely to be achieved only in the long term, immediate interventions are needed to support sex workers, including the funding and scale-up of specialist and sex-worker-led services that can address the multiple and linked health and social care needs that sex workers may face.

## Supporting information

S1 Moose Checklist (DOC)

S1 Fig Sensitivity analysis of unadjusted and adjusted estimates of HIV/STI stratified by police exposure. (TIF)

S2 Fig Sensitivity analysis of unadjusted and adjusted estimates of sexual/physical violence stratified by police exposure. (TIF)

S3 Fig Sensitivity analysis of unadjusted and adjusted estimates of condomless sex stratified by police exposure. (TIF)

S4 Fig Sensitivity analysis of outcome misclassification. (TIF)

S1 Table Quality assessment of quantitative studies. (XLSX)

S2 Table Data used in R for meta-analysis. (XLSX)

S1 Text Systematic review protocol. (DOC)

S2 Text Summary of CERQual assessment. (DOCX)

S3 Text Category themes and sub-themes. (DOCX)

S4 Text All references reviewed as part of qualitative synthesis. (DOCX)

## Abbreviations

cis: cisgender
OR: odds ratio
STI: sexually transmitted infection
trans: transgender
