# Supplementary figures and images for: Associations between sex work laws and sex workers’ health: A systematic review and meta-analysis of quantitative and qualitative studies
Source: PLoS Med. 2018 Dec 11;15(12):e1002680. doi: 10.1371/journal.pmed.1002680 (PMC6289426; doi:10.1371/journal.pmed.1002680)

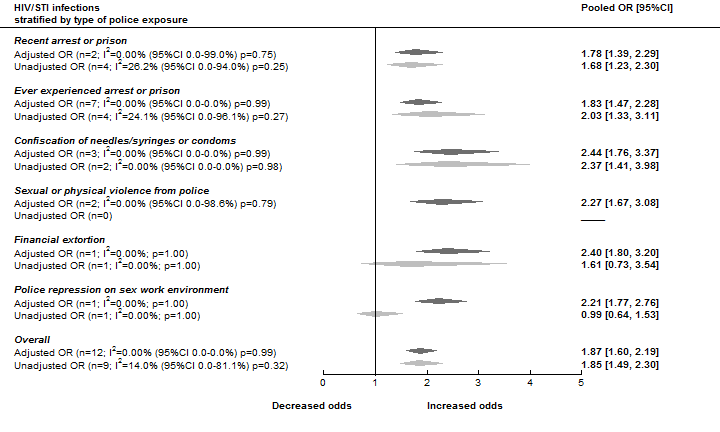

Supplement: S1 Fig — (TIF) [file pmed.1002680.s002.tif]

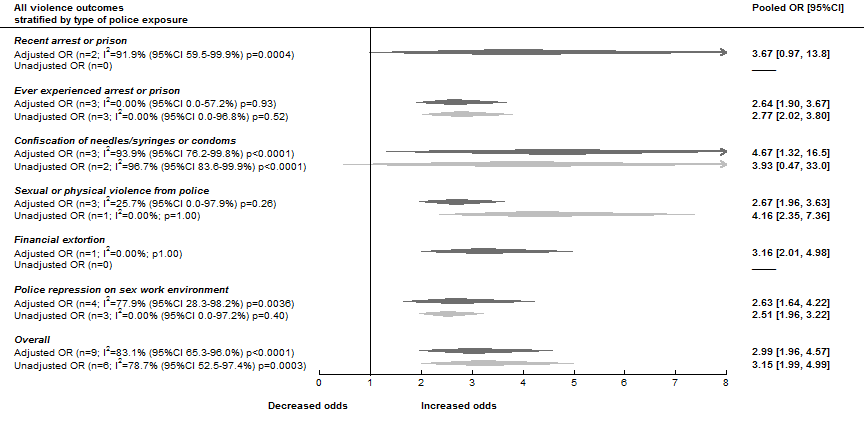

Supplement: S2 Fig — (TIF) [file pmed.1002680.s003.tif]

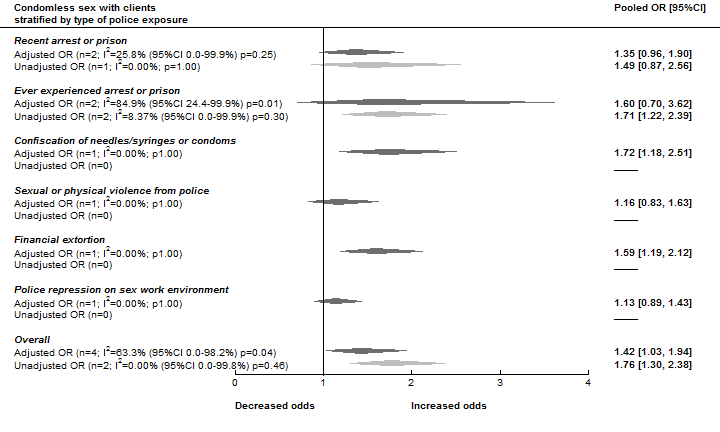

Supplement: S3 Fig — (TIF) [file pmed.1002680.s004.tif]

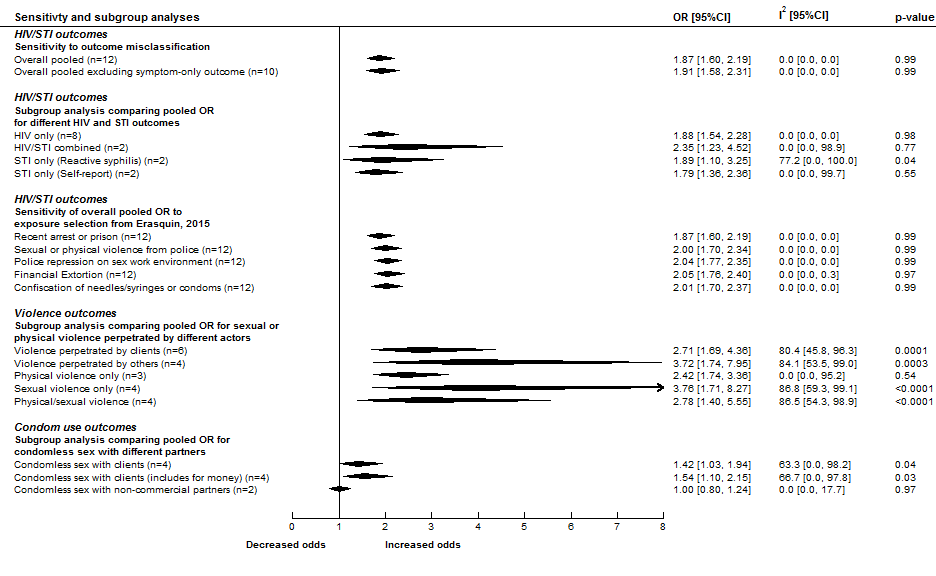

Supplement: S4 Fig — (TIF) [file pmed.1002680.s005.tif]
