# Supplementary material for: Associations between sex work laws and sex workers’ health: A systematic review and meta-analysis of quantitative and qualitative studies
Source: PLoS Med. 2018 Dec 11;15(12):e1002680. doi: 10.1371/journal.pmed.1002680 (PMC6289426; doi:10.1371/journal.pmed.1002680)
Supplement: S1 Text — (DOC) [file pmed.1002680.s008.doc]

**Understanding how criminalisation impacts on sex workers' health:**

**a review of the evidence**

**Why this study is important**

A rich body of social science and ‘grey’ literature documents the harms of criminalisation of sex work on sex workers’ health and safety. ^1-6^ These effects may be direct, by restricting sex workers’ capacity for risk reduction, or they may be indirect, by interacting with other structural determinants of poor health. The threat of police harassment and arrest, for example, reduces time for screening clients and negotiating condom use and displaces sex workers into more isolated and dangerous work locations and can impact directly on risk reduction capacity, for example by rushing screening of clients and negotiation of condom use, or through confiscation of condoms as evidence of sex work. ^1-3^ Meanwhile, criminalisation and policing of sex work reinforces stigma, exacerbates economic and housing insecurity and limits access to health services, while hindering collectivization – all of which, in turn, increase vulnerability to violence, HIV and poor emotional health, and restrict sex workers’ human and citizenship rights.^7, 8^

In line with increasing attention on how social policies and structural factors shape public health, epidemiologists have begun to turn their attention to these questions. Recently, several systematic reviews of the structural correlates of HIV and violence demonstrated that criminalisation, policing and imprisonment were independently and consistently associated with increased risk, across diverse social, economic and cultural contexts. ^9-11^ Linked mathematical modelling estimated that decriminalisation of sex work could reduce the incidence of HIV among sex workers and their clients by up to 46% over a 10-year period. ^10^ Studies in the UK, Russia, Mexico, Argentina, Canada and India have shown the association between imprisonment and policing and increased risk of other sexually transmitted infections (STIs), violence, lower rates of condom use and poor access to services.^9, 12-16^ These data lend statistical support to the larger body of social science and community evidence documenting the ways in which criminalisation impacts on sex workers’ health.

To date, no synthesis of this literature has been undertaken. At a time of increasing political interest in further criminalising sex work, across Europe and elsewhere^17-19^, there is a critical need to bring together this evidence, such that it can be used to maximum effect by activists, academics and practitioners advocating for evidence-based policy that protects sex workers’ health, well-being and broader rights. This research will also be directly relevant to the commissioning, design and evaluation of public health interventions for sex workers.

**What we will do**

We propose to conduct a review of existing public health, social science and ‘grey’ literature to synthesize evidence of: (i) the magnitude of associations between criminalisation, or its removal, and sex workers’ health; and (ii) the processes and pathways through which these effects occur, including in interaction with other structural and social factors such as stigma, housing and collectivisation.

The specific research questions to be answered through the review are:

1. What are the associations between (de)criminalisation of sex work (see definitions below), and the following: violence; STIs, HIV, hepatitis B/C; sexual and reproductive health (e.g. pregnancy, abortion); drug-related harms (e.g. overdose); emotional health; and access to health/social care?
2. What are the processes and pathways through which (de)criminalisation shapes: sex workers’ safety, physical and emotional health; strategies to manage these risks; and access to health and social care; including in interaction with other structural and social factors?

**Methods**

Search strategy

We will conduct a search of the peer-reviewed public health and social science literature via the following databases: Medline, CINAHL, Psych Info, Web of Science and Global Health. We will employ a search strategy that combines the following three domains including appropriate regional terms and spellings, refining where necessary and using truncation and other search devices to capture relevant iterations. Examples of key words to be searched for are listed in Appendix A:

1. Sex work AND
2. Legislation and policing AND
3. Health and access to care including: a) physical and emotional OR access to health and social care/risk reduction

To identify grey and non-indexed peer reviewed literature, we will use a simplified version of the above strategy within the following organisations’ websites: Research for Sex Work; Global Network of Sex Work Projects; TAMPEP; Sex Worker Open University; Sex Workers’ Rights Advocacy Network; International Committee on the Rights of Sex Workers in Europe; International Harm Reduction Association; Open Society Foundations; UNAIDS; HIV/AIDS Alliance; and the Population Council. We will contact academic and community experts, including via the Sex Work Research Hub, to identify additional relevant literature.

Definitions

We will define sex workers as adult cis- and transgender women and men who currently or have ever exchanged sexual services for money, drugs or goods. We will draw on Phoenix’s definition of ‘criminalisation of sex work’ ^20^- “*a model of intervention in which the criminal law is used to manage, control, repress, prohibit or otherwise influence the growth, instance or expression of prostitution*”– but also including in our review the use of non-criminal penalties to target sex workers, such as fines and displacement orders. Criminalisation of sex work will be operationalised to include: harassment, warnings or arrests for soliciting, loitering or kerb-crawling; raids, closure or demolition of sex work venues/areas; civil orders to remove sex workers from certain spaces; confiscation of condoms as evidence of sex work; and confiscation of funds or assets presumed to be proceeds of sex work.

Inclusion criteria

We will include studies that: i) are published in English, Russian or Spanish; and ii) include data specific to the experiences of sex workers. *Additional inclusion criteria for quantitative studies* are that they examine or model associations between (de)criminalisation of sex work and any of the following outcomes: physical or emotional health, (threatened or enacted violence, STIs, HIV, hepatitis B/C, overdose, stress, anxiety, depression); risk practices/management (condom use, sharing needles/syringes, (not) working with others, (not) reporting violence) or access to health and social care services (HIV/STI/hepatitis prevention, testing and treatment; contraception; abortion; opioid substitution therapy and other drug/alcohol services; mental health and counselling; primary and secondary care; gender transitioning care; psychosocial support services; housing; social security). *Additional inclusion criteria for qualitative studies* are that they (i) explore sex workers’ lived experiences of health and safety, risk management and/or accessing health/social care, and (ii) (de)criminalisation and/or policing forms a substantive focus of the article. Inclusion criteria may be further refined depending on the scope of the literature identified and may include for example focussing qualitative literature on outcomes explored in the quantitative synthesis only.

Data synthesis and quality assessment

For quantitative literature, if data are sufficiently homogenous in terms of outcomes and definition of criminalisation, outcome measures will be subject to statistical meta-analysis. Otherwise, data will be synthesized narratively. For qualitative studies, data will be synthesized thematically. We will assess the quality of included studies following methodologies such as the Critical Appraisal Skills Programme (CASP)^21^ and, in the case of qualitative literature, drawing on additional published guidance. ^22^ We will include studies which, at a minimum, use appropriate and ethical study designs and analysis methods, and the conclusions of which are supported by the study’s findings.

Advisory Group

We will convene a virtual advisory group consisting of epidemiologists, sociologists, criminologists and sex workers to advise on the review protocol, conduct, dissemination and strategies for feeding findings into advocacy and policy, to ensure that our interpretation of the review synthesis reflects experiences of the community and the academy, and to maximise its community impact.

**Appendix A: Search terms**

**Search terms**

We will be identifying articles which include at least one term from each of the three domains within their title, abstract or key words:

1. Sex work (sex work* OR prostitut* OR street walker* OR escort* OR rent boy*OR sell sex OR sold sex OR selling sex OR exchanged sex OR exchange sex OR exchanged sex OR sex trade OR commercial sex OR sex industry OR transactional sex OR sexual favour OR bar hostess OR red light district)

AND

1. Legislation and policing
   1. (criminalis* OR criminaliz* OR prohibit* OR decriminalis* OR decriminaliz* OR legalisation OR legalization OR legislat* OR law* OR legal* OR illegal* OR policing OR police* OR enforce* OR criminal justice OR prison* OR penalt* OR fine* OR raid* OR police operation* OR arrest* OR detention* OR detain* OR sanction* OR dispersal order* OR zoning restriction* OR zon* OR convict* OR police record* OR criminal record*)
   2. (anti-social behaviour* OR (police ADJ3 harrass*) OR (police ADJ3 repress*) OR (police ADJ3 warning) OR (police ADJ3 evict*) OR (police ADJ3 engage$) OR (police ADJ3 caution) OR civil order* OR administrative offense OR (police ADJ3 coercion) OR confiscat* OR crime prevention OR brothel closure* OR confiscat* OR loiter* OR solicit* OR support order OR rehabilit* OR (vagrancy ADJ3 act) OR (municipal ADJ3 order) OR by-law OR police repress* OR licens* OR regulation OR mandatory test* OR registration OR forced test* OR condoms as evidence)

AND

1. Health and access to care - searching for terms related to:
   1. physical and emotional health and safety (e.g. violen* OR brutal* OR attack* OR assault* OR rape* OR safety OR security OR protection OR murder OR homicide OR HIV OR sexually transmitted OR chlamydia OR gonorrhoea OR syphilis OR Treponema Pallidum OR trichomonas OR pelvic inflammatory disease OR urinary tract infection* AIDS OR sexual health OR hepatitis OR overdos* OR reproductive health OR pregnan* OR abortion* OR miscarriage* OR mental health OR emotional health OR psychological OR depress* OR anxiet* OR stress OR self-harm OR suicid* OR pyschiatr* OR well-being OR self-esteem OR mental disorder OR self-perception OR therap$
   2. access to health and social care/risk reduction (condom* OR contracept* OR clinic* OR outreach OR hospital* OR health care OR health service* OR psychologist* OR psychiatrist* OR counselling OR needle* OR syringe OR harm reduction OR methadone OR buprenorphine OR opioid substitution OR nalaxone OR gender transition* OR gender reassignment surger* OR hormone treatment* OR hormone therap* OR psychosocial support OR housing OR accommodation OR shelter* OR social services OR social welfare OR social security OR primary care OR GP OR general practi* OR family doctor* OR dentist* OR dental care OR dental treatment OR test* OR treat* OR outreach OR specialist service OR Social care service* OR social care* OR voluntary sector* OR voluntary service* OR voluntary care service* OR community care* OR social service OR safeguarding OR child protection OR evict* OR loans OR access to clean water OR education OR micro-finance).

**MESH TERMS:**

1. Prostitution Sex workers
2. Police, Law enforcement, Legislation (as topic), Criminal Law, Crime, Prisons
3. Health services accessibility, Violence, reproductive health, sexually transmitted diseases, HIV, mental health, Safety, Condoms, harm reduction, Counseling, Opiate substitution therapy
