# Supplementary material for: Associations between sex work laws and sex workers’ health: A systematic review and meta-analysis of quantitative and qualitative studies
Source: PLoS Med. 2018 Dec 11;15(12):e1002680. doi: 10.1371/journal.pmed.1002680 (PMC6289426; doi:10.1371/journal.pmed.1002680)
Supplement: S2 Text — (DOCX) [file pmed.1002680.s009.docx]

**Summary of CERQual assessment**

| **Category** | **Included papers** | **CERQual assessment of confidence in the evidence** | **Explanation of CERQual assessment** |
| --- | --- | --- | --- |
| Disrupted workspaces and protective strategies | (Abel, 2014; Anderson et al., 2016; Armstrong, 2014; Armstrong, 2015; Armstrong, 2016; Baratosy and Wendt, 2017; Benoit et al., 2016; Biradavolu et al., 2009; Brents and Hausbeck, 2005; Cepeda and Nowotny, 2014; Corriveau and Greco, 2014; Ghimire et al., 2011; Goldenberg et al., 2018; Handlovsky et al., 2012; Huang and Pan, 2014; Katsulis et al., 2010; Krusi et al., 2012; Krusi et al., 2016; Krusi et al., 2014; Levy and Jakobsson, 2014; Maher et al., 2015; Maher et al., 2011; Miller, 2002; O'Doherty, 2011; Okal et al., 2011; Pitcher and Wijers, 2014; Pyett and Warr, 1999; Ratinthorn et al., 2009; Rocha-Jimenez et al., 2017; Shannon et al., 2008; Sherman et al., 2015; Simic and Rhodes, 2009) | **High/moderate** | **Moderate to high methodological quality**  *All studies used appropriate design, methods and ethical approach, conclusions reflected findings; a sizeable minority lacked detail over sampling and analysis methods; few studies reflected on relationship between researchers and participants in detail*  **High coherence**  *No findings contradict the review finding; most included studies contribute partially but clearly to this category*  **High adequacy of data**  *33 papers contribute to this category (15 rich data)*  **Moderate to high relevance**  *Wide range of countries, populations & legislative models, but some regions (Africa, Central/South America, Europe) & models (criminalisation of clients, decriminalisation) underrepresented, and few studies with trans and male sex workers* |
| Institutionalising violence, coercion and extortion, and restricting access to justice | (Abel, 2014; Armstrong, 2014; Armstrong, 2015; Armstrong, 2016; Baratosy and Wendt, 2017; Benoit et al., 2016; Biradavolu et al., 2009; Cepeda and Nowotny, 2014; Corriveau and Greco, 2014; Dewey and St Germain, 2014; Ediomo-Ubong, 2012; Foley and Nguer, 2010; Ghimire et al., 2011; Goldenberg et al., 2018; Gulcur and Ilkkaracan, 2002; Huang and Pan, 2014; Karim et al., 1995; Katsulis et al., 2010; Kiernan et al., 2016; Krusi et al., 2016; Krusi et al., 2014; Levy and Jakobsson, 2014; Lutnick and Cohan, 2009; Lyons et al., 2017; Maher et al., 2015; Mayhew et al., 2009; Miller, 2002; Nichols, 2010; O'Doherty, 2011; Okal et al., 2011; Pyett and Warr, 1999; Ratinthorn et al., 2009; Rhodes et al., 2008; Rocha-Jimenez et al., 2017; Scorgie et al., 2013; Sherman et al., 2015; Simic and Rhodes, 2009; Wong et al., 2011) | **High/moderate** | **Moderate to high methodological quality**  *All studies used appropriate design, methods and ethical approach, conclusions reflected findings; a sizeable minority lacked detail over sampling and analysis methods; few studies reflected on relationship between researchers and participants in detail*  **High coherence**  *No findings contradict the review finding; most included studies contribute partially but clearly to this category*  **High adequacy of data**  *39 papers contribute to this category (19 rich data)*  **Moderate to high relevance**  *Very wide range of countries, populations & legislative models, but some regions (Central/South America, Europe) & models (criminalisation of clients, decriminalisation) underrepresented, and few studies with trans and male sex workers* |
| Reproducing multiple stigmas and inequalities | (Baratosy and Wendt, 2017; Benoit et al., 2018; Benoit et al., 2016; Biradavolu et al., 2009; Cepeda and Nowotny, 2014; Corriveau and Greco, 2014; Dewey and St Germain, 2014; Goldenberg et al., 2018; Gulcur and Ilkkaracan, 2002; Katsulis et al., 2010; Krusi et al., 2016; Levy and Jakobsson, 2014; Lyons et al., 2017; Maher et al., 2015; Mayhew et al., 2009; Miller, 2002; Nichols, 2010; Ratinthorn et al., 2009; Rhodes et al., 2008; Rocha-Jimenez et al., 2017; Scorgie et al., 2013; Shannon et al., 2008; Sherman et al., 2015; Simic and Rhodes, 2009; Wong et al., 2011) | **High/moderate** | **High methodological quality**  *All studies used appropriate design, methods and ethical approach, conclusions reflected findings; a small number of studies lacked detail over sampling and analysis methods; half of studies reflected on relationship between researchers and participants in detail*  **High coherence**  *No findings contradict the review finding; most contribute clearly but partially to this category, depending on the population and the inequalities/stigmas they face*  **Moderate to high adequacy of data**  *27 papers contribute to this category (8 rich data)*  **Moderate relevance**  *Good range of countries, populations & legislative models, but some regions (Central/South America, Europe) & models (criminalisation of clients) underrepresented and no studies in decriminalised contexts; few studies with trans and male sex workers* |
| Restricted access to healthcare and social welfare | (Anderson et al., 2016; Baratosy and Wendt, 2017; Benoit et al., 2016; Brents and Hausbeck, 2005; Goldenberg et al., 2018; Gulcur and Ilkkaracan, 2002; Ham and Gerard, 2014; Huang and Pan, 2014; Levy and Jakobsson, 2014; Lutnick and Cohan, 2009; Lyons et al., 2017; Maher et al., 2015; O'Doherty, 2011; Rocha-Jimenez et al., 2017; Scorgie et al., 2013) | **Moderate** | **High to moderate methodological quality**  *All studies used appropriate design, methods and ethical approach, conclusions reflected findings; a few studies lacked detail over sampling and analysis methods; a sizeable minority reflected on relationship between researchers and participants in detail*  **Moderate coherence**  *No findings contradict the review finding; most contribute clearly but partially to this category; however, there is greater focus on HIV/STI testing in regulated contexts, and on broader healthcare in other contexts*  **Moderate adequacy of data**  *18 papers contribute to this category (4 rich data)*  **Moderate relevance**  *Good range of countries, populations & legislative models, but all regions except North America represented by 1-2 studies only; regulation most represented model, no studies in decriminalised contexts; few studies with trans and male sex workers* |

Abel, G. M. 2014. A decade of decriminalization: Sex work 'down under' but not underground. *Criminol Crim Justice,* 14**,** 580-592.

Anderson, S., Shannon, K., Li, J., Lee, Y., Chettiar, J., Goldenberg, S. & Krüsi, A. 2016. Condoms and sexual health education as evidence: impact of criminalization of in-call venues and managers on migrant sex workers access to HIV/STI prevention in a Canadian setting. *BMC International Health & Human Rights,* 16**,** 1-10.

Armstrong, L. 2014. Screening clients in a decriminalised street-based sex industry: Insights into the experiences of New Zealand sex workers. *Australian and New Zealand Journal of Criminology,* 47**,** 207-222.

Armstrong, L. 2015. "Who's the Slut, Who's the Whore?": Street Harassment in the Workplace Among Female Sex Workers in New Zealand. *Feminist Criminology,* 11**,** 285-303.

Armstrong, L. 2016. From law enforcement to protection? Interactions between sex workers and police in a decriminalized street-based sex industry. *British Journal of Criminology,* 57**,** 570-588.

Baratosy, R. & Wendt, S. 2017. "Outdated Laws, Outspoken Whores": Exploring sex work in a criminalised setting. *Women's Studies International Forum,* 62**,** 34-42.

Benoit, C., Jansson, S., Smith, M. & Flagg, J. 2018. Prostitution stigma and its effect on the working conditions, personal lives, and health of sex workers. *Journal of Sex Research,* 55**,** 457-471.

Benoit, C., Smith, M., Jansson, M., Magnus, S., Ouellet, N., Atchison, C., Casey, L., Phillips, R., Reimer, B., Reist, D. & Shaver, F. M. 2016. Lack of Confidence in Police Creates a "Blue Ceiling" for Sex Workers' Safety. *Canadian Public Policy-Analyse De Politiques,* 42**,** 456-468.

Biradavolu, M. R., Burris, S., George, A., Jena, A. & Blankenship, K. M. 2009. Can sex workers regulate police? Learning from an HIV prevention project for sex workers in southern India. *Soc Sci Med,* 68**,** 1541-1547.

Brents, B. G. & Hausbeck, K. 2005. Violence and legalized brothel prostitution in Nevada: examining safety, risk, and prostitution policy. *Journal of Interpersonal Violence,* 20**,** 270-295.

Cepeda, A. & Nowotny, K. M. 2014. A border context of violence: Mexican female sex workers on the U.S.-Mexico border. *Violence against women,* 20**,** 1506-1531.

Corriveau, P. & Greco, C. 2014. Misunderstanding (mis)understandings: Male sex workers and the Canadian criminal code. *Sexuality & Culture: An Interdisciplinary Quarterly,* 18**,** 346-360.

Dewey, S. & St Germain, T. 2014. "It Depends on the Cop:" Street-Based Sex Workers' Perspectives on Police Patrol Officers. *Sexuality Research and Social Policy,* 11**,** 256-270.

Ediomo-Ubong, E. N. 2012. Sex work, drug use and sexual health risks: occupational norms among brothel-based sex workers in a Nigerian city. *African Journal of Drug and Alcohol Studies,* 11**,** 95-105.

Foley, E. E. & Nguer, R. 2010. Courting success in HIV/AIDS prevention: the challenges of addressing aconcentrated epidemic in Senegal. *African Journal of AIDS Research (AJAR),* 9**,** 325-336.

Ghimire, L., Smith, W. C. S., Teijlingen, E. R. v., Dahal, R. & Luitel, N. P. 2011. Reasons for non-use of condoms and self- efficacy among female sex workers: a qualitative study in Nepal. *BMC Women's Health,* 11.

Goldenberg, S. M., Jimenez, T. R., Brouwer, K. C., Miranda, S. M. & Silverman, J. G. 2018. Influence of indoor work environments on health, safety, and human rights among migrant sex workers at the Guatemala-Mexico Border: a call for occupational health and safety interventions. *Bmc International Health and Human Rights,* 18.

Gulcur, L. & Ilkkaracan, P. 2002. The "Natasha" experience: Migrant sex workers from the former Soviet Union and Eastern Europe in Turkey. *Women's Studies International Forum,* 25**,** 411-421.

Ham, J. & Gerard, A. 2014. Strategic in/visibility: Does agency make sex workers invisible? *Criminol Crim Justice,* 14**,** 298-313.

Handlovsky, I., Bungay, V. & Kolar, K. 2012. Condom use as situated in a risk context: women's experiences in the massage parlour industry in Vancouver, Canada. *Culture, Health & Sexuality,* 14**,** 1007-1020.

Huang, Y. Y. & Pan, S. M. 2014. Government crackdown of sex work in China: Responses from female sex workers and implications for their health. *Glob Pub Health,* 9**,** 1067-1079.

Karim, Q. A., Karim, S. S. A., Soldan, K. & Zondi, M. 1995. Reducing the risk of HIV infection among South African sex workers: Socioeconomic and gender barriers. *Am J Pub Health,* 85**,** 1521-1525.

Katsulis, Y., Lopez, V., Durfee, A. & Robillard, A. 2010. Female Sex Workers and the Social Context of Workplace Violence in Tijuana, Mexico. *Medical Anthropology Quarterly,* 24**,** 344-362.

Kiernan, B., Mishori, R. & Masoda, M. 2016. 'There is fear but there is no other work': a preliminary qualitative exploration of the experience of sex workers in eastern Democratic Republic of Congo. *Culture, Health & Sexuality,* 18**,** 237-248.

Krusi, A., Chettiar, J., Ridgway, A., Abbott, J., Strathdee, S. A. & Shannon, K. 2012. Negotiating safety and sexual risk reduction with clients in unsanctioned safer indoor sex work environments: a qualitative study. *Am J Pub Health,* 102**,** 1154-1159.

Krusi, A., Kerr, T., Taylor, C., Rhodes, T. & Shannon, K. 2016. 'They won't change it back in their heads that we're trash': the intersection of sex work-related stigma and evolving policing strategies. *Sociol Health Illn,* 38**,** 1137-50.

Krusi, A., Pacey, K., Bird, L., Taylor, C., Chettiar, J., Allan, S., Bennett, D., Montaner, J. S., Kerr, T. & Shannon, K. 2014. Criminalisation of clients: Reproducing vulnerabilities for violence and poor health among street-based sex workers in Canada - A qualitative study. *BMJ open,* 4 (6) (no pagination).

Levy, J. & Jakobsson, P. 2014. Sweden's abolitionist discourse and law: Effects on the dynamics of Swedish sex work and on the lives of Sweden's sex workers. *Criminology & Criminal Justice: An International Journal,* 14**,** 593-607.

Lutnick, A. & Cohan, D. 2009. Criminalization, legalization or decriminalization of sex work: what female sex workers say in San Francisco, USA. *Reproductive Health Matters,* 17**,** 38-46.

Lyons, T., Krüsi, A., Pierre, L., Kerr, T., Small, W. & Shannon, K. 2017. Negotiating Violence in the Context of Transphobia and Criminalization: The Experiences of Trans Sex Workers in Vancouver, Canada. *Qualitative Health Research,* 27**,** 182-190.

Maher, L., Dixon, T. C., Phlong, P., Mooney-Somers, J., Stein, E. S. & Page, K. 2015. Conflicting Rights: How the Prohibition of Human Trafficking and Sexual Exploitation Infringes the Right to Health of Female Sex Workers in Phnom Penh, Cambodia. *Health & Human Rights: An International Journal,* 17**,** 102-113.

Maher, L., Mooney-Somers, J., Phlong, P., Couture, M.-C., Stein, E., Evans, J., Cockroft, M., Sansothy, N., Nemoto, T. & Page, K. 2011. Selling sex in unsafe spaces: Sex work risk environments in Phnom Penh, Cambodia. *Harm Reduction Journal Vol 8 Nov 2011, ArtID 30,* 8.

Mayhew, S., Collumbien, M., Qureshi, A., Platt, L., Rafiq, N., Faisel, A., Lalji, N. & Hawkes, S. 2009. Protecting the unprotected: mixed-method research on drug use, sex work and rights in Pakistan's fight against HIV/AIDS. (Special Issue: STIs and HIV in Pakistan: from analysis to action.). *Sex Trans Inf,* 85**,** ii31-ii36.

Miller, J. 2002. Violence and coercion in Sri Lanka's commercial sex industry: intersections of gender, sexuality, culture, and the law. *Violence Against Women,* 8**,** 1044-1073.

Nichols, A. 2010. Dance Ponnaya, Dance! Police Abuses Against Transgender Sex Workers in Sri Lanka. *Feminist Criminology,* 5**,** 195-222.

O'Doherty, T. 2011. Criminalization and off-street sex work in Canada. *Canadian Journal of Criminology and Criminal Justice,* 53**,** 217-245.

Okal, J., Chersich, M. F., Tsui, S., Sutherland, E., Temmerman, M. & Luchters, S. 2011. Sexual and physical violence against female sex workers in Kenya: a qualitative enquiry. *AIDS Care,* 23**,** 612-618.

Pitcher, J. & Wijers, M. 2014. The impact of different regulatory models on the labour conditions, safety and welfare of indoor-based sex workers. *Criminol Crim Justice,* 14**,** 549-564.

Pyett, P. & Warr, D. 1999. Women at risk in sex work: strategies for survival. *Journal of Sociology,* 35**,** 183-197.

Ratinthorn, A., Meleis, A. & Sindhu, S. 2009. Trapped in circle of threats: violence against sex workers in Thailand. *Health Care for Women International,* 30**,** 249-269.

Rhodes, T., Simic, M., Baros, S., Platt, L. & Zikic, B. 2008. Police violence and sexual risk among female and transvestite sex workers in Serbia: qualitative study. *BMJ: British Medical Journal (International Edition),* 337**,** 560-563.

Rocha-Jimenez, T., Brouwer, K. C., Silverman, J. G., Morales-Miranda, S. & Goldenberg, S. M. 2017. Exploring the Context and Implementation of Public Health Regulations Governing Sex Work: A Qualitative Study with Migrant Sex Workers in Guatemala. *Journal of Immigrant and Minority Health,* 19**,** 1235-1244.

Scorgie, F., Vasey, K., Harper, E., Richter, M., Nare, P., Maseko, S. & Chersich, M. F. 2013. Human rights abuses and collective resilience among sex workers in four African countries: a qualitative study. *Globalization and health,* 9**,** 33.

Shannon, K., Kerr, T., Allinott, S., Chettiar, J., Shoveller, J. & Tyndall, M. W. 2008. Social and structural violence and power relations in mitigating HIV risk of drug-using women in survival sex work. *Soc Sci Med,* 66**,** 911-921.

Sherman, S. G., Footer, K., Illangasekare, S., Clark, E., Pearson, E. & Decker, M. R. 2015. “What makes you think you have special privileges because you are a police officer?” A qualitative exploration of police's role in the risk environment of female sex workers. *AIDS Care,* 27**,** 473-480.

Simic, M. & Rhodes, T. 2009. Violence, dignity and HIV vulnerability: Street sex work in Serbia. *Sociol Health Illn,* 31**,** 1-16.

Wong, W. C. W., Holroyd, E. & Bingham, A. 2011. Stigma and sex work from the perspective of female sex workers in Hong Kong. *Sociology of Health & Illness,* 33**,** 50-65.
