# Supplementary material for: Associations between sex work laws and sex workers’ health: A systematic review and meta-analysis of quantitative and qualitative studies
Source: PLoS Med. 2018 Dec 11;15(12):e1002680. doi: 10.1371/journal.pmed.1002680 (PMC6289426; doi:10.1371/journal.pmed.1002680)
Supplement: S3 Text — (DOCX) [file pmed.1002680.s010.docx]

**Categories themes and sub-themes**

1. **Disrupted workspaces and safety strategies**

- Enforcement disrupting workplaces
  - Displacement to secluded/unknown places due to (threat of) raids (against sex workers or clients) or venue closures
  - Disrupted work due to police presence/raids increases financial pressures
  - (Reduced) control over workspace in (un)known/(un)populated workplaces
  - (Fewer) opportunities for 3^rd^ party intervention in (un)known/(un)populated workplaces
  - Enhanced sense of safety and control in regulated/unsanctioned indoor environments due to venue-level safety strategies and security systems
- Avoidance of enforcement disrupting workplaces/strategies
  - Having to choose between working safely/together and avoiding arrest
  - Condoms as evidence deters sex workers and venues from carrying/providing condoms
  - (Absence of) legal protections in (un)regulated workplaces
- Screening and negotiations
  - Rushed screening & negotiations due to police presence/raids (partial/full criminalisation)
  - (Lesser) power over negotiations in (un)known/(un)populated workplaces
  - Increased control over work/client negotiations in context of decriminalisation
  - Increased sense of respect/confidence facilitates negotiation with clients (decriminalisation)
  - Laws mandating condom use increase capacity to insist on condom (decriminalisation)

1. **Institutionalising violence, coercion and extortion, and restricting access to justice**

- Threat of arrest and impunity directly facilitates police abuse of power & violence
  - extortion, theft
  - sexual coercion, verbal & physical abuse
  - blaming & shaming
  - forced into acting as informants, risking retaliation
  - legally-sanctioned police raids enable police violence
- Arbitrary arrest/targeting of sex workers regardless of whether committing offense
- Not/being targeted for arrest reduces/exacerbates fear, anxiety and stress
- Reasons for not reporting to police (full/partial criminalisation, regulation)
  - fear of further criminalisation (of self, of clients)
  - fear that they will not take it seriously
  - fear of blame, disclosure
- Experiences of reporting violence
  - further criminalised – fines, false charges, arrest, detention
  - police ‘failure to protect’ – no action taken
  - blame, humiliation
  - further violence, extortion
- Criminalisation and lack of access to justice facilitates impunity for officers and others
- Police responding to reports of violence (informally-unsanctioned, decriminalisation)

1. **Reproducing multiple stigmas and inequalities**

- Disproportionate punishment of sex workers relative to clients
- Drug use
  - Stopped and searched for drugs
  - blame reinforced for sex workers who use drugs
- Transphobia and homophobia
  - verbal, physical and sexual police abuse targeting gender identity/expression
  - intersecting illegality and stigma re. sex work and homosexuality
  - trans sex workers disproportionately targeted and attacked by police
  - forced gendered behaviour as form of police violence against trans sex workers
- Racism and xenophobia
  - Police targeting sex workers of colour
  - Police using racist insults
  - Police not taking reports of violence against Indigenous women seriously
  - Police targeting migrant sex workers in contexts of criminalised clients (Sweden)
  - History of mistrust, reinforced during experiences in sex work, including entrapment
- Economic insecurity
  - Disrupted work, fines
  - Police extortion and theft
- Regulation reproducing inequalities by excluding most marginalised sex workers
- Threat of disclosure & related stigma
  - Mandatory registration (in contexts of regulation)
  - Police approaching/targeting sex workers outside of working hours

**4. Restricted access to health and social care and support**

- Disrupted access to outreach/clinics and peer resources (criminalisation)
  - displacement via police enforcement
  - venue managers/sex workers’ fear of being reported
  - sex workers’ fears of sharing information with peers
- Conditional access to health & support (client criminalisation/sex work as form of violence)
  - Conforming to victim discourse
  - Committing to exiting
  - Not distributing condoms on outreach
- Restricted access to housing/welfare (client criminalisation/sex work as form of violence)
  - Evictions based on brothel-keeping laws/venue closures
- Regulation contexts
  - Mandatory testing overburdens sex workers
  - Registration requirements restrict access to broader health and social care (e.g. health insurance, loans) due to stigma
