# Supplementary material for: Associations between sex work laws and sex workers’ health: A systematic review and meta-analysis of quantitative and qualitative studies
Source: PLoS Med. 2018 Dec 11;15(12):e1002680. doi: 10.1371/journal.pmed.1002680 (PMC6289426; doi:10.1371/journal.pmed.1002680)
Supplement: S4 Text — (DOCX) [file pmed.1002680.s011.docx]

**All other references reviewed as part of the qualitative synthesis**

1. 1. Abel GM, Fitzgerald LJ. ‘The street's got its advantages’: Movement between sectors of the sex industry in a decriminalised environment. Health, Risk & Society. 2012;14(1):7-23.

2. Alexander P. Sex work and health: a question of safety in the workplace. Journal of the American Medical Womens Association. 1998;53(2):77-82.

3. Amaya A, Canaval GE, Viáfara E. Stigma of sexual worker women: influences on health. Colombia Medica. 2005;36:65-74.

4. Anderson S, Jia X, Liu V, Chattier J, Krusi A, Allan S, et al. Violence prevention and municipal licensing of indoor sex work venues in the Greater Vancouver Area: narratives of migrant sex workers, managers and business owners. Culture, Health & Sexuality. 2015;17(7):825-41.

5. Bandewar SVS, Shalini B, Kongelf A, Pisal H, Collumbien M. Considering risk contexts in explaining the paradoxical HIV increase among female sex workers in Mumbai and Thane, India. 2016.

6. Begum S, Hocking JS, Groves J, Fairley CK, Keogh LA. Sex workers talk about sex work: six contradictory characteristics of legalised sex work in Melbourne, Australia. Culture, Health & Sexuality. 2013;15(1):85-100.

7. Benoit C, Jansson S, Smith M, Flagg J. Prostitution stigma and its effect on the working conditions, personal lives, and health of sex workers. Journal of Sex Research. 2018;55(4-5):457-71.

8. Blankenship KM, Koester S. Criminal law, policing policy, and HIV risk in female street sex workers and injection drug users. Journal of Law, Medicine & Ethics. 2002;30(4):548-.

9. Bodkin K, Delahunty-Pike A, O'Shea T. Reducing stigma in healthcare and law enforcement: A novel approach to service provision for street level sex workers. International Journal for Equity in Health. 2015;14 (1) (no pagination)(35).

10. Bungay V, Guta A. Strategies and Challenges in Preventing Violence Against Canadian Indoor Sex Workers. Am J Pub Health. 2018;108(3):393-8.

11. Cange CW, LeBreton M, Saylors K, Billong S, Tamoufe U, Fokam P, et al. Female sex workers' empowerment strategies amid HIV-related socioeconomic vulnerabilities in Cameroon. Culture, Health & Sexuality. 2017;19(10):1053-65.

12. Crago A-L. "Bitches killing the nation": Analyzing the violent state-sponsored repression of sex workers in Zambia, 2004-2008. Signs. 2014;39(2):365-81.

13. Decker MR, Pearson E, Illangasekare SL, Clark E, Sherman SG. Violence against women in sex work and HIV risk implications differ qualitatively by perpetrator. BMC public health. 2013;13:876.

14. Ewen JE. Using the Venom of a Snake for an Antidote: exploring strategies and services for sex workers in preparation for the World Cup 2014. Perspectives in Public Health. 2015;135(4):197-203.

15. Foley EE. Regulating sex work: subjectivity and stigma in Senegal. Culture, Health & Sexuality. 2017;19(1):50-63.

16. Hunt J, Bristowe K, Chidyamatare S, Hardin R. 'They will be afraid to touch you': LGBTI people and sex workers' experiences of accessing healthcare in Zimbabwe - an in-depth qualitative study. 2017.

17. Jayasree AK. Searching for justice for body and self in a coercive environment: sex work in Kerala, India. Reproductive Health Matters. 2004;12(23):58-67.

18. Koon Teh Y. HIV-related needs for safety among male-to-female transsexuals (mak nyah) in Malaysia. SAHARA J: Journal of Social Aspects of HIV/AIDS Research Alliance. 2008;5(4):178-85.

19. Lakkimsetti C. "HIV Is our friend": Prostitution, biopower, and the state in postcolonial India. Signs. 2014;40(1):201-26.

20. Lewis J, Maticka-Tyndale E. Licensing sex work: Public policy and women's lives. Canadian Public Policy-Analyse De Politiques. 2000;26(4):437-49.

21. Lewis J, Maticka-Tyndale E, Shaver F, Schramm H. Managing Risk and Safety on the Job: The Experiences of Canadian Sex Workers. Journal of Psychology & Human Sexuality. 2005;17(1-2):147-67.

22. Lim S, Peitzmeier S, Cange C, Papworth E, LeBreton M, Tamoufe U, et al. Violence against female sex workers in Cameroon: accounts of violence, harm reduction, and potential solutions. (Special Issue: HIV risks and vulnerabilities among key populations in West and Central Africa - evidence to inform HIV prevention, treatment and care.). JAIDS, Journal of Acquired Immune Deficiency Syndromes. 2015;68(Suppl. 2):S241-S7.

23. Lyons T, Krusi A, Pierre L, Small W, Shannon K. The impact of construction and gentrification on an outdoor trans sex work environment: Violence, displacement and policing. Sexualities. 2017;20(8):881-903.

24. Maticka-Tyndale E, Lewis J, Street M. Making a place for escort work: A case study. Journal of Sex Research. 2005;42(1):46-53.

25. Mbonye M, Nalukenge W, Nakamanya S, Nalusiba B, King R, Vandepitte J, et al. Gender inequity in the lives of women involved in sex work in Kampala, Uganda. Journal of the International AIDS Society. 2012;15 (no pagination)(633).

26. Munoz J, Adedimeji A, Alawode O. They bring AIDS to us and say we give it to them': Socio-structural context of female sex workers' vulnerability to HIV infection in Ibadan Nigeria. Sahara J. 2010;7(2):52-61.

27. Nixon K, Downe P, Gorkoff K, Ursel J. The everyday occurrence - Violence in the lives of girls exploited through prostitution. Violence against Women. 2002;8(9):1016-43.

28. Norton-Hawk MA. The counterproductivity of incarcerating female street prostitutes. Deviant Behavior. 2001;22(5):403-17.

29. Okanlawon K, Adebowale AS, Titilayo A. Sexual hazards, life experiences and social circumstances among male sex workers in Nigeria. Culture, Health & Sexuality. 2013;15:22-33.

30. Onyango MA, Adu-Sarkodie Y, Agyarko-Poku T, Asafo MK, Sylvester J, Wondergem P, et al. "It's all about making a life": poverty, HIV, violence, and other vulnerabilities faced by young female sex workers in Kumasi, Ghana. Journal of acquired immune deficiency syndromes (1999). 2015;68:S131-S7.

31. Orchard T, Vale J, Macphail S, Wender C, Oiamo T. "You just have to be smart': spatial practices and subjectivity among women in sex work in London, Ontario. Gender Place and Culture. 2016;23(11):1572-85.

32. Pauw I, Brener L. 'You are just whores - You can't be raped': Barriers to safer sex practices among women street sex workers in Cape Town. Culture, Health and Sexuality. 2003;5(6):465-81.

33. Penfold C, Hunter G, Campbell R, Barham L. Tackling Client Violence in Female Street Prostitution: Inter-agency Working between Outreach Agencies and the Police. Policing & Society. 2004;14(4):365-79.

34. Pyett P, Warr D. Vulnerability on the streets: Female sex workers and HIV risk. AIDS Care. 1997;9(5):539-47.

35. Richter ML, Scorgie F, Chersich MF, Luchters S. 'There are a lot of new people in town: But they are here for soccer, not for business' a qualitative inquiry into the impact of the 2010 soccer world cup on sex work in South Africa. Globalization and Health. 2014;10 (1) (no pagination)(45).

36. Ross BL. Outdoor Brothel Culture: The Un/Making of a Transsexual Stroll in Vancouver's West End, 1975-1984. Journal of Historical Sociology. 2012;25(1):126-50.

37. Rowe J. The Shantusi Project: Surveying HIV and need in the unregulated sex industry: exploring the needs of drug dependent workers in the illegal sex industry. . Drug and Alcohol Review. 2011;30:78-9.

38. Sanchez LE. Boundaries of legitimacy: Sex, violence, citizenship, and community in a local sexual economy. Law and Social Inquiry-Journal of the American Bar Foundation. 1997;22(3):543-80.

39. Sanders T. The risks of street prostitution: Punters, police and protesters. Urban Studies. 2004;41(9):1703-17.

40. Sweeney L-A, FitzGerald S. A case for a health promotion framework: the psychosocial experiences of female, migrant sex workers in Ireland. International Journal of Migration, Health & Social Care. 2017;13(4):419-31.

41. Twizelimana D, Muula AS. Hiv and aids risk perception among sex workers in semi-urban blantyre, Malawi. Tanzania Journal of Health Research. 2015;17(3).

42. Wojcicki JM, Malala J. Condom use, power and HIV/AIDS risk: Sex-workers bargain for survival in Hillbrow/Joubert Park/Berea, Johannesburg. Social Science and Medicine. 2001;53(1):99-121.

43. Zeng H, Zhang L, Zhao Y, Liu H, Guo H, Wang Y, et al. HIV prevention among street-based sex workers (SSWs) in Chongqing, China: interviews with SSWs, clients and healthcare providers. Health & Social Care in the Community. 2016;24(6):E173-E80.

44. Zeng H, Zhao Y, Meng SY, Tang XJ, Guo H, Wang Y, et al. Exploring HIV Prevention Strategies among Street-Based Female Sex Workers in Chongqing, China. International Journal of Environmental Research and Public Health. 2015;12(1):855-70.

45. Zhang X-D, Kelly-Hanku A, Chai J-J, Luo J, Temmerman M, Luchters S. Sexual and reproductive health risks amongst female adolescents who use amphetamine-type stimulants and sell sex: A qualitative inquiry in Yunnan, China. Harm Reduction Journal Vol 12 Dec 2015, ArtID 34. 2015;12.
